# Challenges and advances for glioma therapy based on inorganic nanoparticles

**DOI:** 10.1016/j.mtbio.2023.100673

**Published:** 2023-06-01

**Authors:** Die Hu, Miao Xia, Linxuan Wu, Hanmeng Liu, Zhigang Chen, Hefeng Xu, Chuan He, Jian Wen, Xiaoqian Xu

**Affiliations:** a Key Laboratory of Cell Biology, Ministry of Public Health and Key Laboratory of Medical Cell Biology, Ministry of Education, China Medical University, Shenyang, 110122, China; b Department of General Surgery, The Fourth Affiliated Hospital of China Medical University, Shenyang, 110032, China; c Department of Laboratory Medicine, The First Hospital of China Medical University, Shenyang, 110001, China

**Keywords:** Glioma, Inorganic nanoparticles, BBB penetration, Biocompatibility, Diagnosis and treatment

## Abstract

Glioma is one of the most serious central nervous system diseases, with high mortality and poor prognosis. Despite the continuous development of existing treatment methods, the median survival time of glioma patients is still only 15 months. The main treatment difficulties are the invasive growth of glioma and the obstruction of the blood-brain barrier (BBB) to drugs. With rapid advancements in nanotechnology, inorganic nanoparticles (INPs) have shown favourable application prospects in the diagnosis and treatment of glioma. Due to their extraordinary intrinsic features, INPs can be easily fabricated, while doping with other elements and surface modification by biological ligands can be used to enhance BBB penetration, targeted delivery and biocompatibility. Guided glioma theranostics with INPs can improve and enhance the efficacy of traditional methods such as chemotherapy, radiotherapy and gene therapy. New strategies, such as immunotherapy, photothermal and photodynamic therapy, magnetic hyperthermia therapy, and multifunctional inorganic nanoplatforms, have also been facilitated by INPs. This review emphasizes the current state of research and clinical applications of INPs, including glioma targeting and BBB penetration enhancement methods, *in vivo* and *in vitro* biocompatibility, and diagnostic and treatment strategies. As such, it provides insights for the development of novel glioma treatment strategies.

## Graphical abstract

Glioma treatment is facing to many challenges. Theranostic inorganic nanoparticles is becoming popular in recent years, as they can provide opportunities to combat glioma through different diagnosis and treatment strategies with satisfied biocompatibilities. Summarize of the current progresses would provide exceptional insights for a new generation of nanomaterials to the management of glioma.

**Figure:**
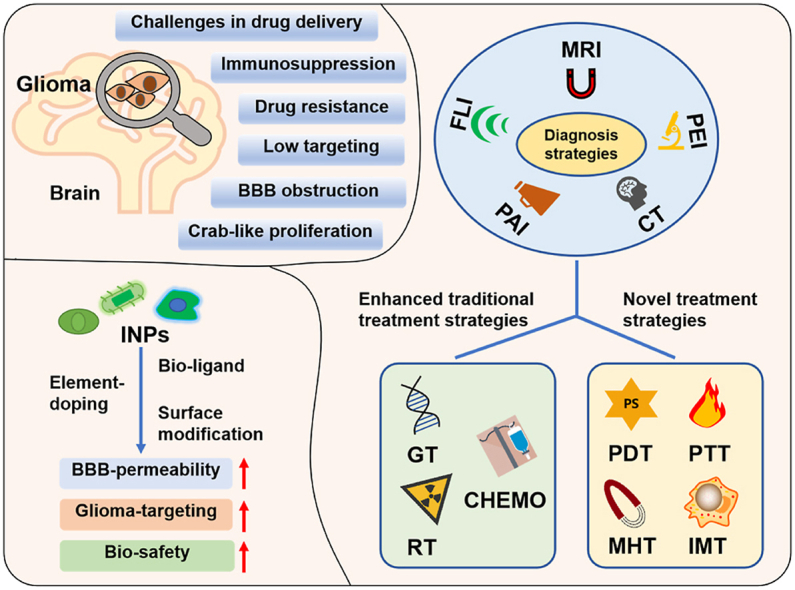

## 1 Introduction

Glioma is a type of brain tumor that originates from glial stem cells or progenitor cells. It accounts for 30% of all primary brain tumors and 80% of all malignant brain tumors. Glioma is also the most common malignant tumor in the central nervous system (CNS) and its patients have a poor prognosis and a high risk of relapse and death. Currently, the main clinical treatment modes are surgical resection, radiotherapy, chemotherapy [1]. However, because of the particularity of the intracranial structure and the pathological characteristics of glioma, current treatments often lead to high recurrence rates, susceptibility to drug resistance, large side effects, and poor prognosis. Hence, new technological strategies are urgently needed to overcome these difficulties and provide improved glioma treatment strategies.

**Fig. 1:**
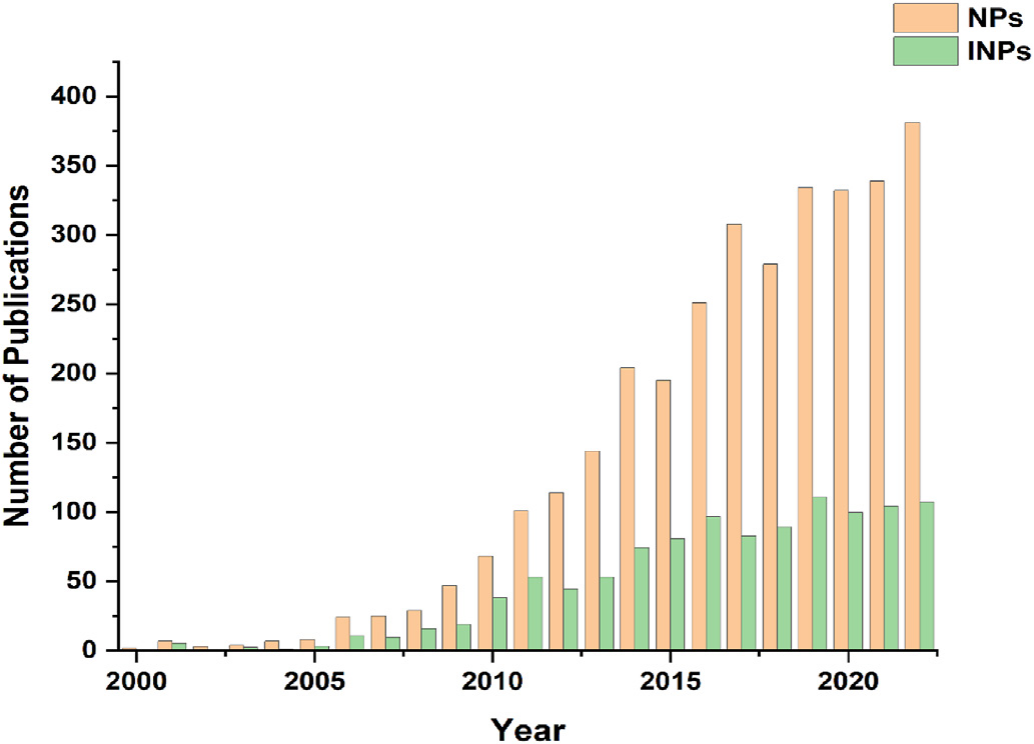
The bibliometric analysis of nanomaterials used in the diagnosis and treatment of glioma. Data source from the Web of Science Core Collection (2000–2022).

With the development of nanotechnologies, nanomaterials have been extensively explored in the field of biomedical applications, including the diagnosis and treatment of glioma. Research on the application of nanoparticles (NPs) in the diagnosis and treatment of glioma has been ongoing since the late 20th century. The increasing number of articles over the past 20 years were summarized in Fig. 1, which indicates that approaches brought about by nanotechnology have attracted much attention. The reviews in the past five years mainly focus on the following aspects, including NPs based targeted drug delivery systems to overcome BBB, advances of nanomaterials in brain diseases but not limited to glioma, the prospect of NPs as carrier-loaded Temozolomide (TMZ) targeting therapy for glioblastoma (GBM), and the application of nanomedicine in glioma immunotherapy or gene therapy [[2], [3], [4], [5], [6], [7]].

Among them, INPs have also experienced considerable development. As shown in Fig. 1, the application research of INPs in the diagnosis and treatment of glioma also shows a trend of development year by year, with an annual circulation of about 100 articles in the past five years. Initially, the research mainly focused on the application of magnetic nanoparticles (MNPs) for magnetic resonance imaging (MRI) and gradually expanded the research scope of inorganic nanomaterials, including but not limited to metal nanomaterials, graphene-based nanomaterials, mesoporous silica nanoparticles (MSNs), upconversion nanoparticles, and quantum dots [8]. For instance, Norouzi et al. [9] reviewed and discussed the multiple applications of Au NPs in the diagnosis and treatment of gliomas from 2004 to 2020, including chemotherapy, phototherapy, gene therapy, MRI, Photoacoustic imaging (PAI), surface-enhanced Raman scattering (SERS). They discussed the cellular uptake and pharmacokinetic differences of gold-based NPs (GNPs) of different shapes, and crossing BBB capabilities of GNPs of different types, as well as the possible impact of varying tumor models on NPs studies *in vivo*, paving the way for future clinical studies. In another work, Choi et al. [10] reviewed previous studies using high atomic number (high-Z) metal NPs in GBM radiotherapy between 2005 and 2020 to showcase the potential of high-Z metal NPs application in the radiosensitization of GBM. Verma et al. [11] introduced studies in which MNPs, gold nanorods (GNRs), and carbon nanotubes (CNTs) have been applied for hyperthermia in GBM from 1996 to 2014. They introduced the diverse synthesis methods of various INPs and discussed their application in hyperthermia ablation and enhanced drug delivery in GBM. To date, research progress of glioma diagnosis and treatment based on INPs has been updated rapidly.

Generally, the physical and chemical properties of inorganic elements, such as the quantum size effect, surface effect, small size effect, macroscopic quantum tunnelling effect and dielectric domain effect, can be obtained by nanoscale construction [12,13]. Consequently, a series of properties propitious to biological applications can be generated, especially for tumor diagnosis and treatment. These include efficient endocytosis with low cytotoxicity, an enhanced tumor permeability and retention effect, high optical imaging efficiency, and tumor ablation effects that can be excited by physical means, such as light and nuclear magnetism. The increasing biomedical research into INPs is providing new strategies and opportunities for the diagnosis and intervention of gliomas [14,15]. The main inorganic elements currently employed for nanoparticle fabrication related to glioma research are gold (Au), silver (Ag), copper (Cu), cadmium (Cd), zinc (Zn), titanium (Ti), molybdenum (Mo), lanthanum (La), gadolinium (Gd), superparamagnetic iron oxides, graphene (GPE), mesoporous silicon, and carbon (C). INPs fabricated by these elements have been widely used in tumor therapies, such as for tumor imaging, improved drug target delivery, synergistic therapy, prolonged drug cycling, reduced cytotoxicity, and the utilization of photosensitizers (PSs) in Photothermal Therapy (PTT) to obtain safer and more effective therapeutic effects [[16], [17], [18]].

**Fig. 2:**
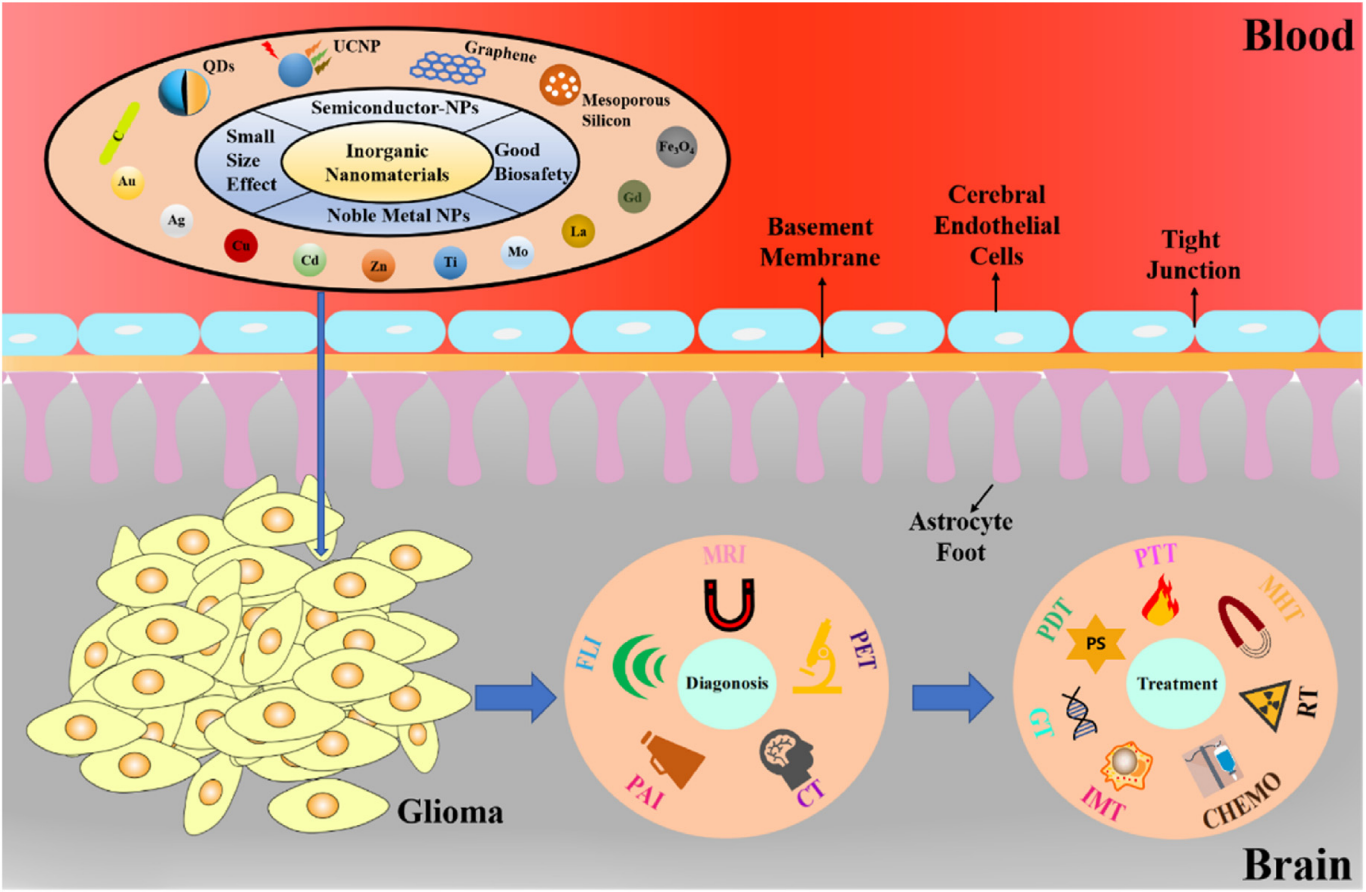
Schematic illustration of BBB-crossing inorganic nanoplatforms used for efficient glioma theranostics.

Nevertheless, there are few comprehensive and systematic reviews of glioma diagnosis and treatment based on all types of INPs mentioned above. The advantages of our work lie in the selection of representative research results in the past five years, as well as the unique and systematic summary of the latest progress and new glioma treatment strategies of INPs. First, the staging and typing of gliomas are introduced according to the World Health Organization's (WHO) clinical guidelines. Then, the characteristics of BBB structures at different stages of glioma and the commonly used targeting ligands are summarized. Furthermore, the biocompatibility of different INPs is compared and analyzed. Finally, nanotechnology applications specific to glioma, such as various imaging modalities, new strategies and improvements in glioma treatment based on INPs, are comprehensively compared and discussed. Fig. 2 is a schematic illustration of the BBB-crossing inorganic nanoplatforms used for highly efficient glioma theranostics.

## 2 Classification of glioma and limitations of treatment

Gliomas can be classified into many types according to malignancy grades. WHO classification of CNS tumors is always the gold standard in clinical work. According to the disease progression, they are mainly categorized into four grades (1–4) [19]. WHO grade 1 includes the least aggressive tumors, which are curable if they can be surgically removed. In contrast, WHO grade 4 tumors are highly malignant and hold the highest mortality rate and shortest survival time. In the newest 5th edition of the guidelines, gliomas are classified into five different families according to their grade of malignancy: adult-type diffuse gliomas, paediatric-type diffuse low-grade gliomas, paediatric-type diffuse high-grade gliomas, circumscribed astrocytic gliomas, and ependymomas [20]. Their gene alterations are summarized in Table 1. Among them, the more aggressive gliomas are the diffuse tumors, which are characterised by rapid growth. Diffuse gliomas, which account for the majority of glioma patients, are characterized by tumor cells invading the nervous system individually or in groups, and present diffuse infiltrating growth in the parenchyma of the CNS [21]. Moreover, this particular pattern of diffuse cell invasion is rare in other tumors [22]. In the traditional classification, diffuse gliomas are histologically classified as astrocytomas (astrocytic), oligodendrocytomas (oligodendrocytic), and mixed gliomas. In the 2021 WHO classification of CNS tumors, diffuse gliomas are divided into adult and paediatric types for the first time. This classification is mainly based on the clinical characteristics of the tumor distributions in different age groups. This does not mean that adult-type gliomas do not occur in children. Paediatric gliomas are classified into two types: low-grade with a good prognosis and high-grade with a very poor prognosis. The name of circumscribed astrocytic gliomas is relative to the diffuse gliomas, whose growth pattern is more limited; however, there is still a risk of invasion. The pathogenesis of gliomas is very complex; however, the recognized root causes are mainly gene mutations *in vivo*, as well as abnormal cell signalling pathways and functional disorders. The common mutations mainly include IDH, H3, BRAF. A detailed summary of the latest 2021 WHO glioma grading its associated genes markers/genetic alterations is provided in Table 1. Among the primary brain tumors, the GBM type undoubtedly has the highest degree of malignancy. GBM is considered to be the most aggressive and highly invasive type since it can quickly spread to any part of the brain. Thus, it is classified as grade 4, which has the worst prognosis [23]. Currently, obstacles to the treatment of glioma include the intricacy of brain tissue, acquired resistance owing to chemotherapy, the heterogeneity and aggressiveness of the tumor, and difficulty in detecting malignant tissue margins. Surgical excision is always the first choice. However, most gliomas possess crab-like infiltrative growth properties and some tumors are always located in brain areas that dominate important functions; therefore, it is difficult to determine malignancies from normal brain tissue [24]. Consequently, total surgical excision of gliomas is almost impossible due to their aggressive behaviour. Even after substantial resection, gliomas can still recur locally within a few centimetres of the resection margin [25]. TMZ as the first-line chemotherapy drug for glioma, is prone to drug resistance [26]. Treatment by radiotherapy must be considered carefully, since severe or even irreversible damage to the CNS can be generated [27]. In addition, the presence of the BBB structure limits the implementation of most treatments. One of the greatest challenges to the development of effective drugs for the treatment of glioma disease is their capacity to effectively penetrate the BBB.

**Table 1:** Classification and grading of gliomas [20].

Glioma	Classification	Name	Genes markers/Genetic alterations	WHO grade
Diffuse gliomas	Adult-type diffuse gliomas	Astrocytoma	IDH1, IDH2, ATRX, TP53, CDKN2A/B	2/3/4
		Oligodendroglioma	IDH1, IDH2, 1p/19q, TERT promoter, CIC, FUBP1, NOTCH1	2/3
		Glioblastoma	IDH-wildtype, TERT promoter, chromosomes 7/10, EGFR	4
	Paediatric-type diffuse low-grade gliomas	Diffuse astrocytoma∗	MYB, MYBL1	1
		Angiocentric glioma	MYB	1
		Polymorphous low-grade neuroepithelial tumor of the young∗	BRAF, FGFR family	1
		Diffuse low-grade glioma	FGFR1, BRAF	NA
	Paediatric-type diffuse high-grade gliomas	Diffuse midline glioma	H3 K27, TP53, ACVR1, PDGFRA, EGFR, EZHIP	4
		Diffuse hemispheric glioma∗	H3 G34, TP53, ATRX	4
		Diffuse paediatric-type high-grade glioma∗	IDH-wildtype, H3-wildtype, PDGFRA, MYCN, EGFR (methylome)	4
		Infant-type hemispheric glioma∗	NTRK family, ALK, ROS, MET	NA
Non-diffuse gliomas	Circumscribed astrocytic gliomas	Pilocytic astrocytoma	KIAA1549-BRAF, BRAF, NF1	
		High-grade astrocytoma with piloid features∗	BRAF, NF1, ATRX, CDKN2A/B (methylome)	NA
		Pleomorphic xanthoastrocytoma	BRAF, CDKN2A/B	
		Subependymal giant cell astrocytoma	TSC1, TSC2	
		Chordoid glioma	PRKCA	
		Astroblastoma∗	MN1	NA
	Ependymal tumors	Supratentorial ependymoma∗	ZFTA, RELA, YAP1, MAML2	2/3
		Posterior fossa ependymoma∗	H3 K27me3, EZHIP (methylome)	2/3
		Spinal ependymoma∗	NF2, MYCN	NA
		Myxopapillary ependymoma		2
		Subependymoma		

The Arabic numerals 1–4 are used to represent the tumoral grades. Those ∗ labelled are newly added tumor types, some ∗ labelled tumors have not been clearly graded, and the rest can refer to the 2016 fourth-edition.

## 3 INPs-based solutions for efficient glioma treatment

A high drug utilization ratio is critical for glioma treatment, as low drug utilization often leads to the drug's side effects outweighing its efficacy. Drug delivery to glioma can be categorized into two main areas: bypassing BBB and crossing BBB. Denzil et al. [28] provide a concise overview of how therapeutic drugs can be delivered to the CNS without establishing BBB-crossing pathways. Therapeutic methods that bypass BBB have problems, including slow drug distribution, rapid drug elimination through active transport, and extremely low penetration into the brain parenchyma [29]. There are few studies on the biological application of INPs in the bypass BBB pathways. This paper mainly discusses glioma treatment strategies through the BBB-crossing pathways based on INPs. In general, the efficiency of drugs used to treat glioma by BBB-crossing methods is dependent on both their BBB permeability ability and their targeting ratio to glioma [30]. Nanomedicine holds promise for the development of successful strategies to penetrate the BBB and target glioma. In this section, we discuss the INPs-based solutions to the main challenges for guaranteeing the drug utilization ratio.

In addition, it is also worth mentioning that, benefit from the responsive properties to some physical factors, including laser, optoacoustic signal and magnetic field, INPs can also enhance the penetration of BBB and combat glioma. In this article, INPs used as photoacoustic/magnetic/fluorescent responsive nanoprobes for molecular imaging, see Sections 5 and 6.3. On the other hand, INPs used for phototherapy/magnetothermal therapy due to the characteristics of the optical/magnetic response of inorganic elements were reviewed in Section 6.2.

### 3.1 Structure of BBB and challenges during the progression of glioma

The BBB is constituted of brain capillary endothelial cells (BCECs), pericytes, astrocytes, tight junctions, neurons, and the basement membrane [31], which is a diffusion barrier that prevents the entry of substances from the blood into the brain [32]. The transendothelial electrical resistance of the BBB is estimated to be 8000 ​W/cm^2^ [33], which permits the BBB to separate blood from brain tissue while also selectively inhibiting the entry of certain toxic macromolecules into the brain circulation [34,35].

During the progression of glioma, the integrity of BBB structure also changes. In the early pathological period, BBB structure is typically intact, while it is gradually disrupted with tumor progression. However, some tumor lesions are encapsulated by endothelial cells, which can still prevent drugs from arriving at the tumor area [36,37]. Therefore, BBB penetration efficiency should be considered during all stages of glioma development. In addition, a drug's ability to target glioma cells is also important in improving the drug utilization ratio.

The BBB penetration ratio is the first challenge for glioma treatment. Many therapies have proven efficient in killing glioma cells *in vitro*; however, due to their inability to penetrate the BBB [38], systemic dosing fails to stop tumor progression *in vivo*, while excessive doses can cause general toxicity to the main organs. In addition, the poor solubility and short half-life of many therapeutic medicines in blood circulation limit their penetration of the BBB. In the aspect of molecular permeability [39], molecules greater than 400 ​Da cannot pass through the BBB, especially in highly water-soluble settings, unless a suitable specific transporter is present. Fat-soluble small molecules tend to be more BBB-permeable. For instance, the DNA-alkylating drug TMZ, which has poor solubility in water, crosses the BBB easily and is one of the few available treatments for GBM [40,41]. There is evidence that albumin barely crosses the intact BBB at all [42], so drugs that bind to plasma proteins may only cross the BBB through specific transporters. Some non-nanomedicine methods have been used clinically to solve the challenges of crossing the BBB and targeting gliomas. For example, arterial mannitol perfusion has been used to enhance the delivery of chemotherapy drugs to gliomas, since hypertonic solutions cause rapid contraction of BCECs, temporarily increase the endocytosis rate, and destroy tight junctions [43]. Another very promising and less-invasive approach to improve drug delivery is transcranial-focused ultrasound [[44], [45], [46]]; however, repeated stimulations are needed. Despite this, the survival rate for glioma is still very low and more efficient therapeutic methods are urgently required.

### 3.2 Fabrication strategies of theranostic INPs for combating glioma

A large number of INPs have been extensively studied for glioma treatment, due to their excellent physical properties and different targeting strategies. According to the functions, they can be used for drug delivery, imaging, phototherapy, radiotherapy, gene therapy, immunotherapy and magnetic hyperthermia.

#### 3.2.1 BBB-crossing strategies based on INPs

Some INPs exhibit the ability to freely penetrate the BBB and passively target gliomas. For example, Sun et al. [47] synthesized a novel fluorescent carbon dot (CD-Asp) using d-glucose and l-aspartate via a straightforward thermolysis route. *In vivo* imaging of C6 glioma-bearing mice injected with CD-Asp via the tail vein was performed. The results indicated that CD-Asp could freely cross the BBB and accumulate at glioma tissue because of its high enhanced permeability and retention efficiency (EPR). Therefore, CD-Asp is an ideal sensitizer to guide non-invasive glioma fluorescent imaging *in vivo*.

**Fig. 3:**
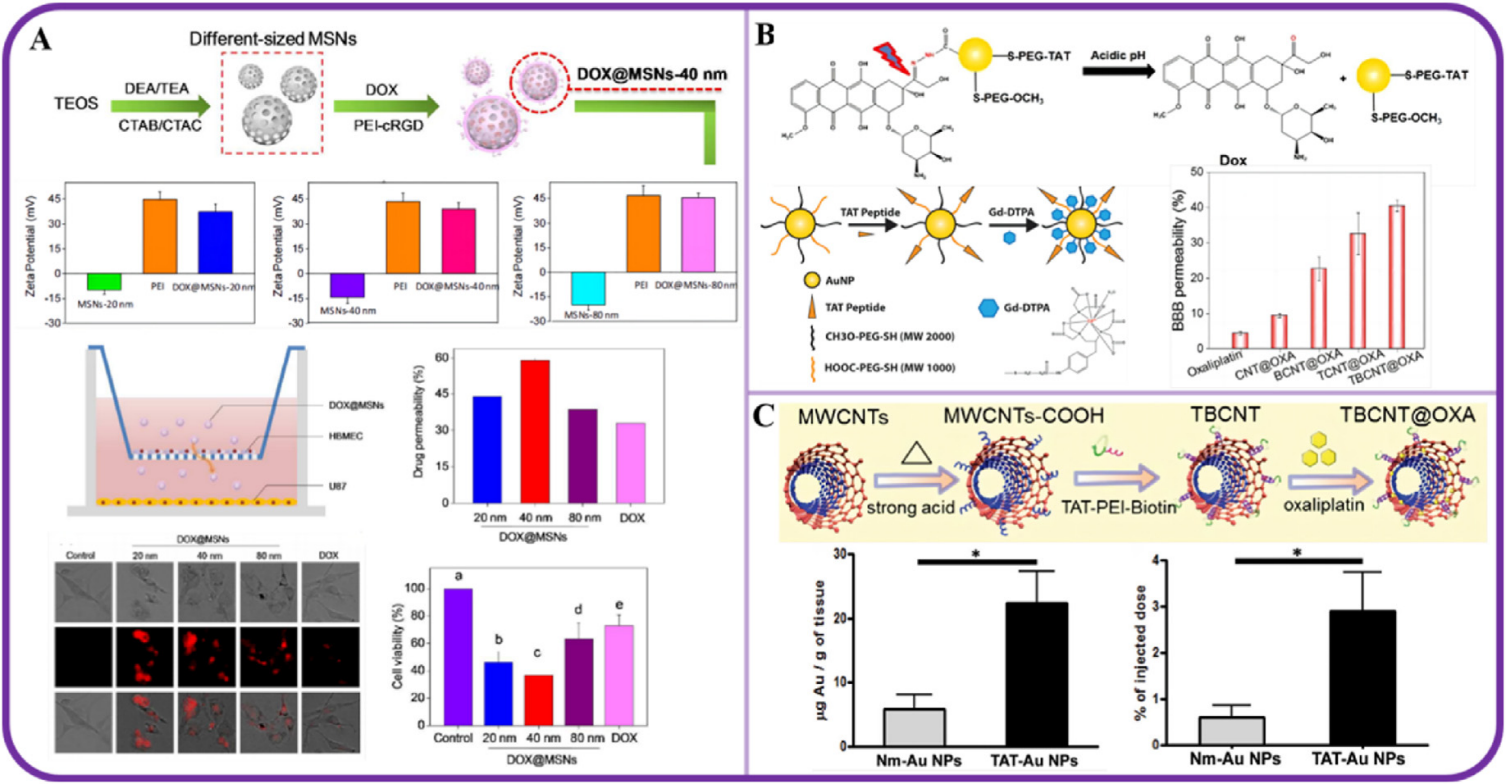
**BBB-crossing strategies based on INPs used for efficient glioma treatment.**A.DOX@MSNs; B. TAT-Au NP-Dox/TAT-Au NP-Gd; C. O-MWNTs-PEG-ANG. Panel A is adapted with permission from Ref. [48], copyright 2016 ACS Applied Materials & Interfaces; Panel B is adapted with permission from Ref. [58], copyright 2014 Small. Panel C is adapted with permission from Ref. [59], copyright 2019 Dalton Transactions.

Size, charge and shape are crucial influences on the uptake and BBB penetration abilities of INPs. In Chen's work [48], the size of MSNs was controlled by adjusting the hydrolysis rate and degree of condensation of the reactants, then loaded with doxorubicin (DOX) and modified with (polyethyleneimine) PEI-cRGD tumor-targeting polymers to enhance the anti-glioma effect (Fig. 3A). Human brain microvascular endothelial cells (HBMVEC) and human brain astrocytoma (U87) glioma cells co-culture were established as an *in vitro* BBB model to assess the BBB-penetrating ability of DOX@MSNs. Fluorescence imaging revealed the uptake of permeable drugs. DOX@MSNs showed higher BBB permeability and cellular uptake than free DOX. Furthermore, among DOX@MSNs with three particle sizes (20 ​nm, 40 ​nm, and 80 ​nm), DOX@MSNs of 40 ​nm particle size exhibited the maximum BBB transport rate and drug toxicity toward glioma cells. In view of this, the wrong particle size may be detrimental to BBB permeability. So, carefully altering the particle size of MSNs may be an effective strategy for combating GBM and overcoming the BBB barrier effect. Unsurprisingly, the surface electricity of INPs plays a crucial role in their brain delivery. Generally, positively charged nanoparticles are more accessible to cells, as adsorption-mediated transcytosis is triggered by electrostatic interactions between cationic targeting components and the negatively charged membranes of BCECs [49]. As described above, Chen [48] changed the charge of DOX@MSN INPs from negative to positive by PEI-cRGD modification. These changes enhanced the cellular uptake and stability of these nanoparticles in comparison with free MSNs. Shape is also an important influence on INPs uptake and penetration into the brain. Eswaramoorthy et al. [50] synthesized iron oxide (Fe_3_O_4_) nanoparticles with different morphologies (spheres, spindles, biconcaves, and nanotubes) and coated them with a fluorescent carbon layer derived from glucose (Fe_3_O_4_@C). The biconcave-shaped nanoparticles (Fe_3_O_4_@CBC) exhibited the highest U87MG cellular uptake ratio. *In vivo* studies further showed that the biconcave nanostructures localized predominantly in the nuclei of the brain cells of mice, whereas nanotube (Fe_3_O_4_@CNT) localized mostly in the cytoplasm. Particle shape affects cellular uptake due to shape-dependent cell surface adhesion [51] and endocytic pathways [52,53]. However, as the nuclear entry pathways of INPs are generally different from those of their cellular entry [54], it cannot be concluded that there is a direct relationship between shape and entry pathways. In a word, the biconcave morphology of Fe_3_O_4_@C enables nuclear-specific drug delivery to brain cells, thereby enhancing their enzymatic activity and corresponding gene expression. Nanotubes can be utilized to deliver molecules specifically into the cytoplasm. Hence, INPs with different morphologies can be selected for drug loading at different brain sites, or have different mechanisms of action and exert different functions.

Moreover, therapeutic efficiency is highly correlated with surface modification ligands, which can determine how nanoparticles cross the BBB and target glioma tissues. A classic example is polyethylene glycol (PEG)-coated Fe_3_O_4_ NPs [55]. A PEG coating on the surface can reduce non-specific protein adsorption and decrease reticuloendothelial system (RES) uptake of Fe_3_O_4_ NPs, thereby obtaining a longer circulation time and increasing the potential for BBB penetration.

In addition, studies have confirmed that surface modification by cell-penetrating peptides (CPP) can be an effective strategy for enhancing BBB penetration efficiency [56]. For instance, the *trans*-activating transcriptional activator (TAT) derived from HIV is used to deliver a wide range of cargoes across lipophilic barriers and can increase the permeability of brain endothelial cells by inhibiting occludin expression and cleaving occludin via matrix metalloproteinase-9 [57]. As described in the literature [58,59], by modification of the TAT peptide on the surface of Au and Carbon-based INPs, BBB permeability can be increased from 9.4% to 40.4% and from 0.6% to 2.9%, respectively (Fig. 3B and C).

#### 3.2.2 Active targeting strategies based on INPs

More importantly, a series of active targeting ligands have been selected based on specific receptors and BBB transporters, which can also be used to enhance the targeting ability of the INP delivery system [[60], [61], [62], [63]]. At present, studies have reported INP delivery systems modified with Angiopep-2 (ANG), lactoferrin (Lf), transferrin (Tf), asparagine-glycine-arginine (NGR) peptide, and RGD peptide dimer (RGD2). Common INP delivery systems and their related target groups for glioma diagnosis and treatment are briefly described in Table 2.

**Table 2:** Summary of the BBB-penetrating strategies of INPs.

Inorganic nanoplatform			Connection methods	BBB-crossing ligand	Proposed transporter(s)	Target-ing glioma	BBB permeability	Main cargo (es)	Ref.
Drug loading	Au	TAT-Au NP-Dox/TAT-Au NP-Gd	Hydrazone	TAT peptide	None	NO	0.6% → 2.9%	DOX	[58]
		An-PEG-DOX-AuNPs	Hydrazone	ANG	LRP1	mono-targeting	Not mentioned	DOX	[64]
	Fe	FeGd-HN@LF/RGD2	EDC/NHS	Lf/RGD2	Lf receptor/integrin αvβ3	dual-targeting	13.6 ​± ​1.9% → 41.0 ​± ​0.9%	CDDP	[65]
		Lf-Cur-PDNCs	EDC/NHS	Lf	Lf receptor	mono-targeting	fourfold	Cur	[66]
	Si	MNP-MSN-PLGA-Tf NPs	EDC/NHS	Tf	Tf receptor	dual-targeting	8 ​→ ​33	DOX and PTX	[67]
		DOX-MNP-MSN-PF-127-Tf	EDC/NHS	Tf	Tf receptor	mono-targeting	4296 ​→ ​56,747	DOX	[68]
		ANG-PTX-PLGA-DOX-MSNs-75	EDC/NHS	ANG	LRP1	mono-targeting	2627 ​→ ​7497	DOX and PTX	[69]
		DOX@MSNs	EDC	cRGD peptide	ανβ3 integrin	mono-targeting	32.8% → 59.2%	DOX	[48]
		DOX@MSN–SS–iRGD&1 ​MT	PEG	iRGD peptide	None	NO	Not Mentioned	DOX	[70]
	C	O-MWNTs-PEG-ANG	PEG	ANG	LRP1	dual-targeting	Not Mentioned	DOX	[71]
		TBCNT@OXA	None	TAT-PEI-Biotin copolymer	None	No	9.4% → 40.4%	OXA	[59]
Diagnosis/Treatment	Au	^131^I–Au PENPs-CTX	PEG、EDC	Chlorotoxin (CTX)	MMP2	mono-targeting	Not Mentioned	None	[72]
	Cd	QD-MPA	NHS	T7 peptide	Tf receptor	mono-targeting	Not Mentioned	None	[73]
		NGR-PEG-QDs	PEG	NGR peptide	CD13	mono-targeting	Not Mentioned	None	[74]
		QD-Apt	PEG	aptamer 32	EGFRvIII	mono-targeting	Not Mentioned	None	[75]
	La (Er)	NaErF4:Ce@NaAF4@NaLuF4 DCNPs	SH	ANG	LRP1	mono-targeting	2.2-fold	None	[76]
	Si	RVG-PEG-AuNR@SiO2	MAL-PEG-NHS	RVG29 peptide	Nicotinic AchR	mono-targeting	Not Mentioned	None	[77]

∗EDC represents for 1- ethyl-3-(3-dimethylaminopropyl) carbodiimide hydrochloride; NHS represents for N-hydroxysuccinimide; SH represents for SH seal; MAL represents for maleimide; CDDP represents for cisplatin; Cur represents for curcumin; PTX represents for paclitaxel; OXA represents for oxaliplatin; MMP2 represents for matrix metalloprote-inase 2; EGFRvIII represents for epidermal growth factor receptor variant III.

Based on the active targeting strategies and the alternative ligands, numerous mono- and dual-targeting nanoplatforms were fabricated for the integrated diagnosis and treatment of glioma at different stages.

##### 3.2.2.1 Mono-targeting INPs

**Fig. 4:**
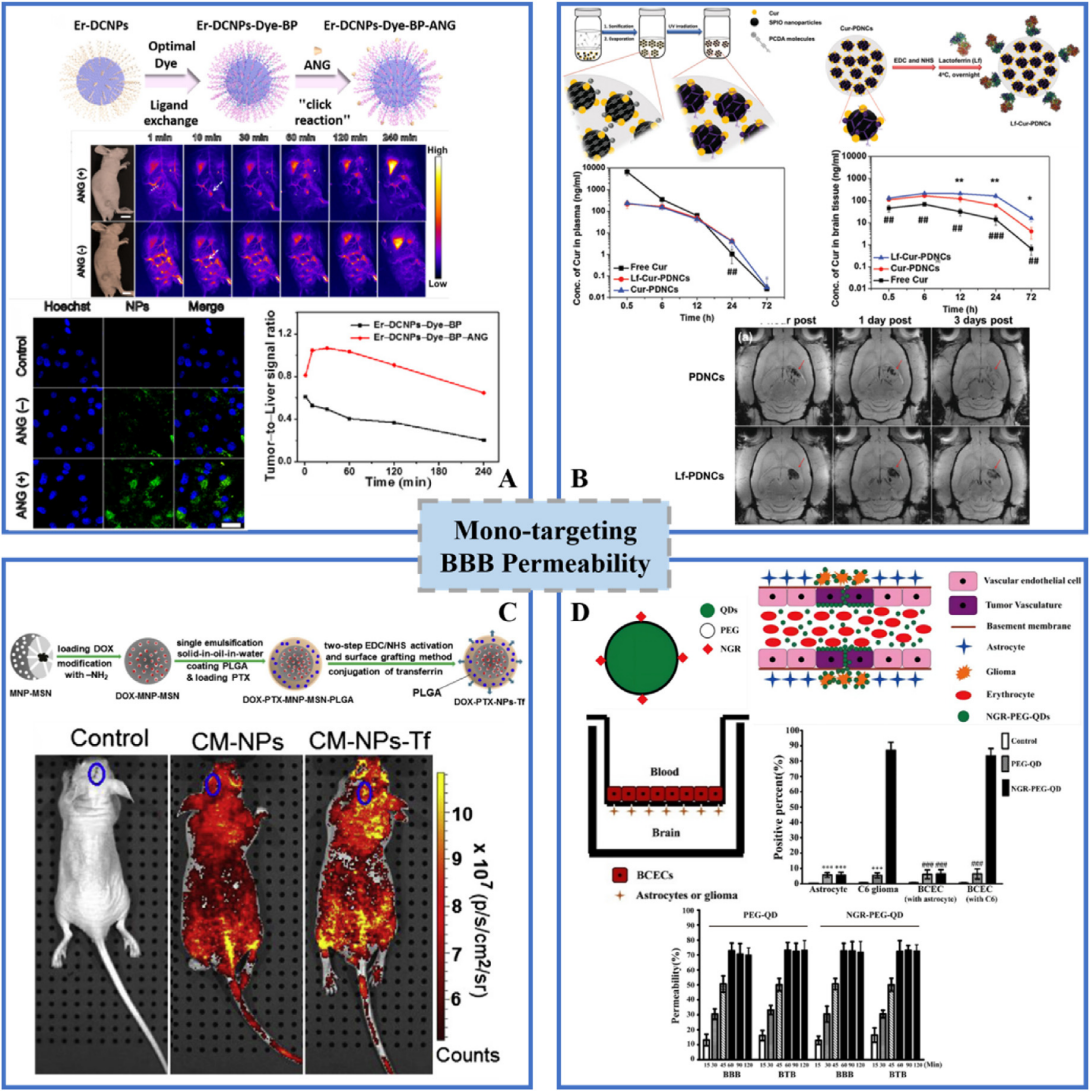
**Mono-targeting INPs used for efficient glioma treatment.** A. Er-DCNPs-Dye-BP-ANG; B. Lf-Cur-PDNCs; C. MNP-MSN-PLGA-Tf NPs; D. NGR-PEG-QDs. Panel A is adapted with permission from Ref. [76], copyright 2020 Nano Today. Panel B is adapted with permission from Ref. [66], copyright ​2016 Advanced Healthcare Materials. Panel C is adapted with permission from Ref. [67], copyright 2013 Biomaterials. Panel D is adapted with permission from Ref. [74], copyright 2016 Nanomedicine: Nanotechnology, Biology, and Medicine.

ANG targeting ligands can enhance glioma treatment efficacy through interactions with low-density lipoprotein (LRP) receptor-related proteins. Gao and colleagues [76] investigated NaErF4:Ce@NaAF4@NaLuF4 down-conversion nanoparticles with Dye-brush polymer and angiopep-2 peptide (Er-DCNPs-Dye-BP-ANG) for use in imaging-guided surgery of orthotopic glioma (Fig. 4A). They employed focused ultrasound sonication, which can temporarily control the opening of the BBB and bind microvesicles to enhance the delivery of nanoprobes to gliomas [[78], [79], [80]]. The study demonstrated that U87 ​cells incubated with Er-DCNPs-Dye-BP-ANG had stronger fluorescence than cells from the Er-DCNPs-Dye-BP group. Then, Er-DCNPs-Dye-BP-ANG was injected intravenously for fluorescence imaging of subcutaneously transplanted gliomas. The tumor brightness of the ANG (+) group was much higher than that of the ANG (−) group. Moreover, the tumor-liver fluorescence ratio increased 2.2-fold in the presence of ANG. In conclusion, these results demonstrate the significant potential of Er-DCNPs-Dye-BP-ANG for fluorescence imaging-guided glioma surgery due to the excellent targeting ability of the ANG peptide to the LRP receptor.

Many studies have found that lactoferrin receptor (LfR) is not only present on the BBB, but also on the cell surface of GBM [81]. Therefore, INPs coated by Lf-targeting ligands can enhance transport of nanodrugs across the BBB and accumulation at the glioma site via receptor-mediated transcytosis and endocytosis [82,83]. A magnetic polydiacetylene nanocarrier (PDNC) delivery system modified with Lf ligands was used to confirm that Lf ligands can increase BBB transport rates (4-fold) while targeting gliomas (Fig. 4B) [66]. Similarly, Tf has also been widely applied to enhance the cellular uptake of INPs, since Tf-receptors are overexpressed in the brain capillary endothelium and glioma cells, involved in receptor-mediated transcytosis [84,85]. Tf-conjugated magnetic silica PLGA nanoparticles (MNP-MSN-PLGA-Tf NPs) loaded with DOX and PTX are a promising carrier of dual drugs for brain glioma treatment, as they increase transport across the BBB and blood-tumor barrier (BTB; Fig. 4C) [67].

Another interesting study synthesized a unique nanoprobe by combining biotinylated NGR peptide with avidin PEG-coated CdSe/ZnS quantum dots (QDs) [74]. Although the heavy metal Cd^2+^ in the CdSe core is toxic to organisms, biocompatibility can be enhanced by coating the surfaces of CdSe/ZnS core/shell QDs with ZnS shells and PEG [86,87]. CD13 is a biomarker expressed in tumor blood vessels [88]. Compared with other tumor types, it has higher expression in gliomas and the tumor-associated neovascularization stage [89]. So, functional NGR sequences could target CD13-overexpressing tumors [90,91]. Based on this, a back-to-back BBB/BTB system was developed by seeding BCEC on the upper side of a filter membrane and astrocytes or C6 glioma cells on the lower side (Fig. 4D). Rapid infiltration occurred mainly in the first hour and then stabilized. After 5 ​h, more than 80% of the cells were labelled by the NGR-PEG-QDs in the BTB model. In summary, this work revealed that NGR-PEG-QDs could cross the BBB and target CD13-overexpressing gliomas. Hence, NGR-PEG-QDs can be used for fluorescence imaging to target glioma and tumor vascular systems.

##### 3.2.2.2 Dual-targeting INPs

**Fig. 5:**
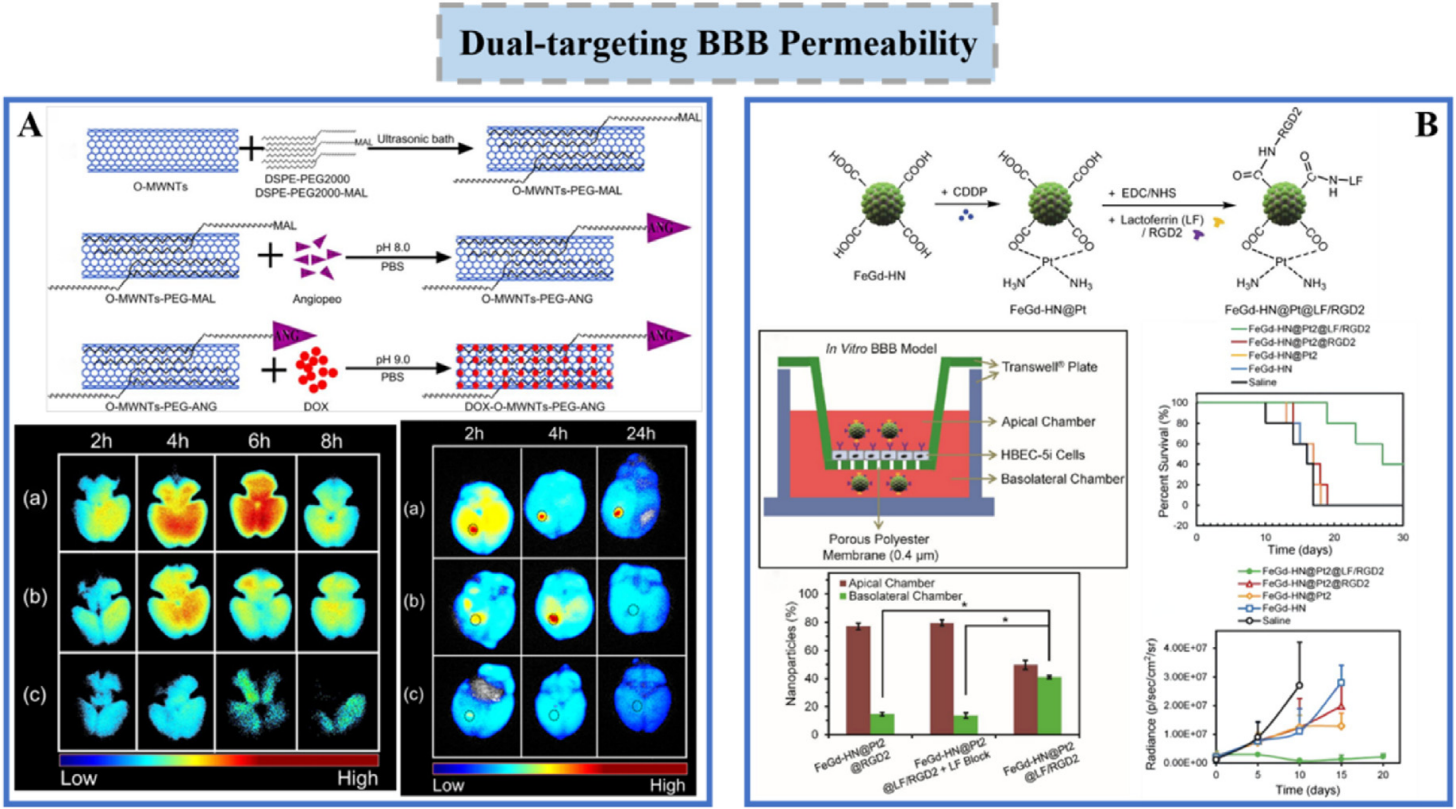
**Dual-targeting INPs used for efficient glioma treatment.** A. TBCNT@OXA; B. FeGd-HN@Pt@LF/RGD2. Panel A is adapted with permission from Ref. [71], copyright 2012 Biomaterials. Panel B is adapted with permission from Ref. [65], copyright 2018 ACS Nano.

Dual-targeting inorganic nanosystems have more efficient BBB penetration and glioma targeting abilities, providing more efficient diagnosis and treatment for early-stage glioma patients with a relatively intact BBB structure. As a case in point, Jiang et al. [71] developed a dual-targeting drug delivery system based on PEGylated oxidized multi-walled carbon nanotubes (O-MWNTs) connected with Angiopep-2 (O-MWNTs-PEG-ANG) for the treatment of brain gliomas (Fig. 5A). O-MWNTs could accumulate in tumors and loaded DOX as a drug carrier. Brain fluorescence images showed that DOX-O-MWNTs-PEG (b) could be distributed in the brain more efficiently than DOX (c) and that DOX-O-MWNTs-PEG-ANG (a) could also enhance the distribution of drugs in the brain. In addition, fluorescence imaging of glioma-bearing brains indicated that DOX-O-MWNTs-PEG could obviously accumulate on gliomas and that DOX-O-MWNTs-PEG-ANG could further enhance glioma targeting. Accordingly, the combination of O-MWNTs-PEG and ANG constitutes an ideal dual-targeting drug delivery system for the diagnosis and treatment of early glioma patients with an intact BBB.

Similarly, CDDP-loaded Fe_3_O_4_/Gd_2_O_3_ hybrid nanoparticles with a conjugation of LF and RGD2 (FeGd-HN@Pt@LF/RGD2) were exploited by Chen's group [65]. FeGd-HN@Pt@LF/RGD2 were designed to cross the BBB by LF receptor-mediated transcytosis and be internalized into cancer cells via integrin αvβ3 (RGD2 receptor) binding, where Fe^2+^, Fe^3+^ and CDDP were released after endosomal uptake and degradation. Fe^2+^ and Fe^3+^ can directly participate in the Fenton reaction, while CDDP can indirectly produce H_2_O_2_ to accelerate it. The Fenton reaction accelerates the generation of reactive oxygen species (ROS), which induces cancer cell death. As shown in Fig. 5B, LF-mediated transcytosis and the use of RGD2 were crucial for the transportability of nanoparticles across the BBB, increasing it from 13.6 ​± ​1.9% to 41.0 ​± ​0.9%. The results demonstrate that FeGd-HN@Pt2@LF/RGD2 nanoparticles have a high Fenton efficacy on orthotopic gliomas.

In summary, a variety of factors (particle size, electrical properties, shape, surface modification, etc.) can influence the BBB-penetrating and tumor-accumulation abilities of INPs. In addition, anchoring antibodies and peptides to nanoparticles can enhance these abilities by interacting with receptors or transporter proteins on endothelial cells. All these factors must be carefully tuned to yield the most efficient inorganic nanoplatforms for the diagnosis and treatment of glioma.

## 4 Biocompatibility evaluations of INPs used for glioma treatment

Toxicity assessment of INPs used for glioma treatment is indispensable before biological application. Unlike other cancer treatments, due to structural and functional complexity of the brain, drugs used for glioma should be considered the avoidance of both neurotoxicity and systemic toxicity. The conventional method is to determine the *in vitro* neurotoxicity of INPs by measuring their biocompatibility with brain microvascular endothelial cells (BMECs), astrocytes and so on. *In vivo* neurotoxicity is evaluated by studying the BBB's integrity or the cognitive and behavioural levels of experimental animals. This section systematically reviews the cell-dependent toxicity, neurotoxicity, major organ damage, biodistribution and clearance of INPs involved in glioma diagnosis and treatment from both *in vivo* and *in vitro* perspectives.

### 4.1 *In vitro* biocompatibility

On cellular level, it is required to consider the influence of INPs on the integrity of the BBB; at least, the toxicity to BMECs, astrocytes and neurons. As summarized in Table 3, various INPs have been extensively studied in both normal nerve cell lines, as well as different glioma cell lines at different conditions to mimick physiological environment.

**Table 3:** Cytotoxicity of INPs used for glioma treatment.

Glioma cell type	Inorganic nanoplatform	Glioma cell safety concentration	Normal cell line (safety concentration)	Incubation time	Ref.
U87 (human brain astrocytoma)	MNP-MSN-PLGA-Tf NPs	100 ​μg/mL	bEnd.3 (100 ​μg/mL)	96 ​h	[67]
	GNS-ICG-BSA	0.5 ​mg/mL	bEnd.3 (0.5 ​mg/mL)	48 ​h	[95]
U251 (human glioblastoma)	L-/D-Cys-CdSe/CdS DRs	50 ​μg/mL	NHA (75 ​μg/mL)	24 ​h	[99]
BV2 (mouse microglia)	GQDs@CCM	200 ​μg/mL	GMI-R1 (200 ​μg/mL)/Rat astrocytes cells (200 ​μg/mL)	24 ​h	[100]
C6 (rat glioma cells)	O-MWNTs-PEG-ANG	100 ​μg/mL	BCEC (100 ​μg/mL)	24 ​h	[71]
	NGR-PEG-QDs	20 ​nM	BCECs, astrocytes (20 ​nM)	96 ​h	[74]
GL261 (mouse glioma cells)	Au–OMV	200 ​μg/mL	bEnd.3 (200 ​μg/mL)/C8D1A (200 ​μg/mL)	24 ​h	[101]

The cytotoxicity of INPs after entering the cell can be mainly determined according to particle size and surface charges. The efficiency of endocytosis uptake is highly dependent on the size of the INPs. Generally, smaller particles enter cells more easily. Therefore, smaller INPs have better biocompatibility, which is beneficial to glioma diagnosis and treatment. Taking the widely employed iron oxide magnetic nanoparticles (IONPs) as an example, Chekhonin et al. [92] investigated the relationship between particle size and toxicity to human glioma cells (U251). They found that within 24 ​h incubation, there was no significant cytotoxicity and no difference between IONPs with different hydrodynamic diameters, even at 10^−3^ ​mol/L concentration. However, after 48 ​h, 40-nm IONPs showed better biocompatibility than 80-nm ones. As a result, it is crucial to tailor the size of INPs to optimise biocompatibility and therapeutic effects on glioma.

In addition to size, the surface charge of nanoparticles affects biocompatibility. It has been demonstrated that positively charged nanoparticles are more easily internalized into cells than neutral and negatively charged ones [93]. This can subsequently affect the endocytosis, passive targeting ratio and cytotoxicity, as described in Section 3.2. Lu's group [94] prepared cetuximab (C225)-encapsulated core-shell Fe_3_O_4_@Au magnetic nanoparticles (Fe_3_O_4_@Au–C225 composite-targeted MNPs). C225 is a monoclonal antibody targeting the EGFR, which is overexpressed in cancer cells. The adsorption of C225 shifted the zeta potential of INPs to a positive charge, enhancing their interactions with glioma cells and improving the internalization efficiency. Of all the concentrations tested, 0.5 ​mg Fe/mL was considered the optimal cell-safe concentration that ensures the killing of glioma cells without damaging normal cells. Ultimately, the dual magnetic and photothermal action mediated by Fe_3_O_4_ @Au–C225 composite-targeted MNPs showed remarkably improved synergistic therapeutic effects in glioma treatment.

**Fig. 6:**
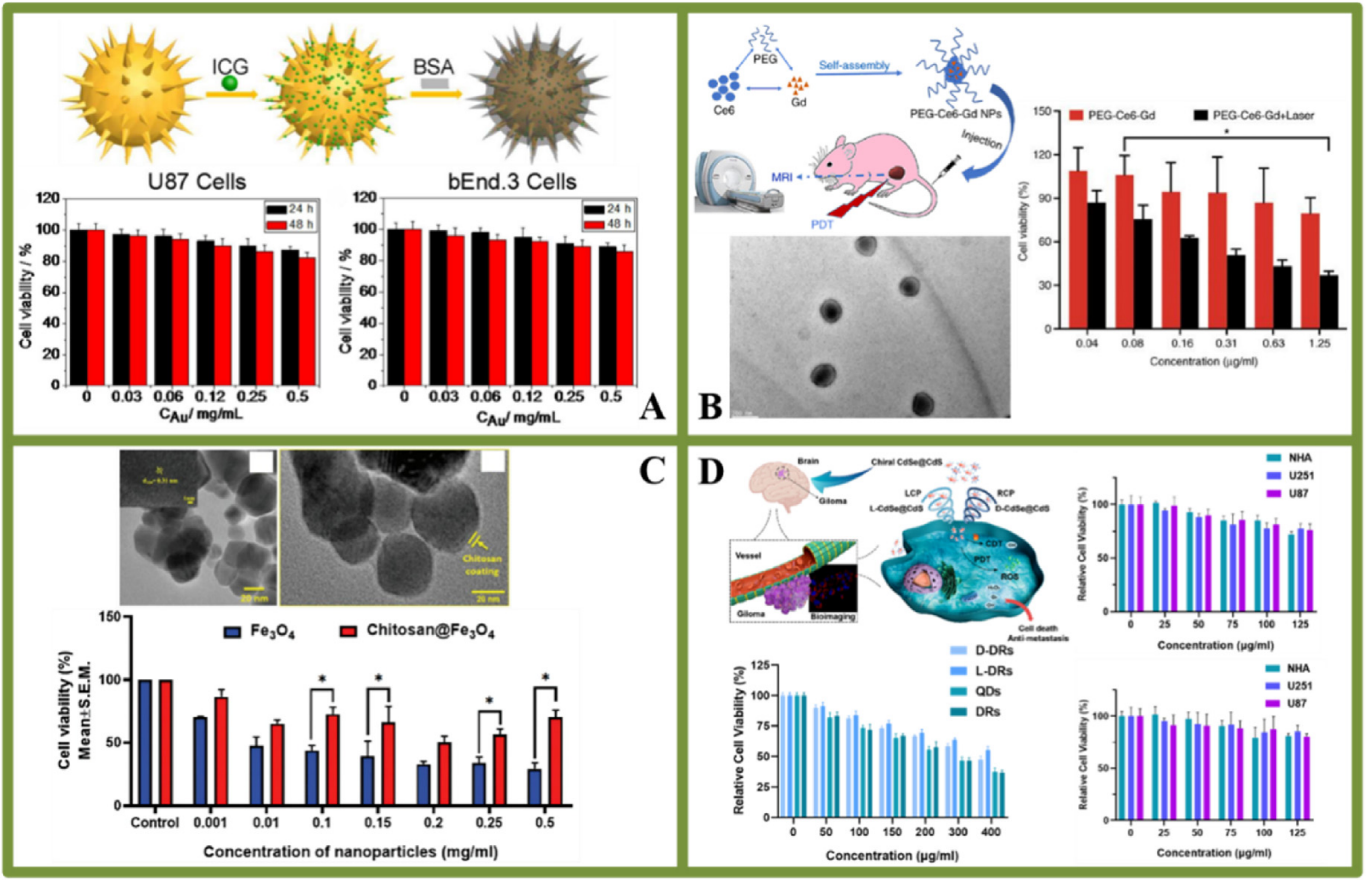
**Cytotoxicity of INPs used for glioma treatment.** A. GNS-ICG-BSA; B. PEG-Ce6-Gd NPs; C. Chitosan-coated Fe_3_O_4_ NPs; D. D-and L-Cys-CdSe/CdS DRs; Panel A is adapted with permission from Ref. [95], copyright 2017 Nanoscale. Panel B is adapted with permission from Ref. [96], copyright 2021 Oncology Reports. Panel C is adapted with permission from Ref. [97], copyright 2021 Biomaterials Science. Panel D is adapted with permission from Ref. [99], copyright 2023 Materials and Design.

Currently, coating and surface modification are the strategies most commonly employed for increasing the biocompatibility of INPs used in the diagnosis and treatment of glioma. Widely used surface modification molecules include bovine serum albumin (BSA), PEG, chitosan, chiral cysteine (Cys) and mercaptopropionic acid (MPA). For example, Wang et al. [95] prepared a non-toxic nanoprobe from gold nanostar-indocyanine green (ICG) incorporated in the BSA (GNS-ICG-BSA) to achieve SERS imaging-guided *in vitro* PTT in U87 glioma cells (Fig. 6A). BSA surface modification can prevent ICG-encoded GNS aggregation under physiological conditions, ensuring excellent stability and biocompatibility of the nanoprobe. Cytotoxicity evaluation of U87 glioma cells and bEnd.3 normal microvascular endothelial cells revealed that GNS-ICG-BSA SERS nanoprobes have satisfactory biocompatibility and low cytotoxicity, even at concentrations of up to 0.5 ​mg/mL. Furthermore, Xu et al. [96] synthesized hydrophilic PEG-chlorin e6 (Ce6)-chelated gadolinium-ion (Gd^3+^) nanoparticles (PEG-Ce6-Gd NPs) via a chelation and self-assembly process (Fig. 6B). PEG-modified nanoparticles present favourable biocompatibility and water solubility, prolonged circulation times and enhanced accumulation in tumor sites via the EPR effect. Biocompatibility results illustrate that proper cytotoxicity make PEG-Ce6-coated Gd-based NPs to be promising glioma nano-agents for photodynamic therapy (PDT) and contrast-enhanced MRI diagnosis. Another classic example is the chitosan-coated Fe_3_O_4_ nanoparticles with high heating efficiency performance developed by Kuanr and colleagues [97] (Fig. 6C). The colloidal stability of Fe_3_O_4_ NPs in a physiological medium can be improved by surface modification using biocompatible chitosan polymers [98]. Notably, the C6 glioma cells treated with chitosan-coated Fe_3_O_4_ NPs displayed significantly lower overall cytotoxicity than that of bare Fe_3_O_4_ NPs, ranging from 0 to 0.5 ​mg/mL over a 24-h testing period. Finally, complete glioma ablation was achieved at a relatively low alternating magnetic field and frequency that was within the clinical safety range. Similarly, Xu et al. [99] synthesized L-Cys and D-Cys modified CdSe/CdS DRs (dot-in-rods) via the post-ligand-exchange method, which exhibited fluorescence imaging-guided photodynamic/chemodynamic therapeutic effects in brain glioma (Fig. 6D). The cellular viability of U251 and U87 human glioma cell lines was maintained at > 90% after 24 ​h incubation with 50 ​μg/mL D-/L-Cys-CdSe/CdS DRs. In addition, chiral CdSe/CdS DRs showed lower toxicity to NHA (normal human astrocytes) than glioma cells in the low concentration range (0–75 ​μg/mL). After that, the cytotoxicity of MPA-modified DRs and QDs (sphere), as well as of L-Cys- and D-Cys-modified DRs, was assessed in U251 ​cells. The results illustrate that the chiral-cysteine coverage strategy can further improve the biocompatibility of CdSe-based QRs and QDs.

According to the needs of glioma diagnosis and treatment (imaging, optical therapy, drug delivery, etc.), we should not only select materials based on the characteristics of their inorganic elements but also consider improving their potential for targeting, reducing toxicity and being used in different modification strategies. This will promote the preparation of corresponding nanocomplexes with diagnostic and treatment functions.

### 4.2 *In vivo* compatibility

In biomedical applications against glioma, INPs are usually delivered into glioma animal models by intravenous or orthotopic injection. INPs generally circulate in the bloodstream and enter the major organs before being cleared by the metabolic organs.

**Fig. 7:**
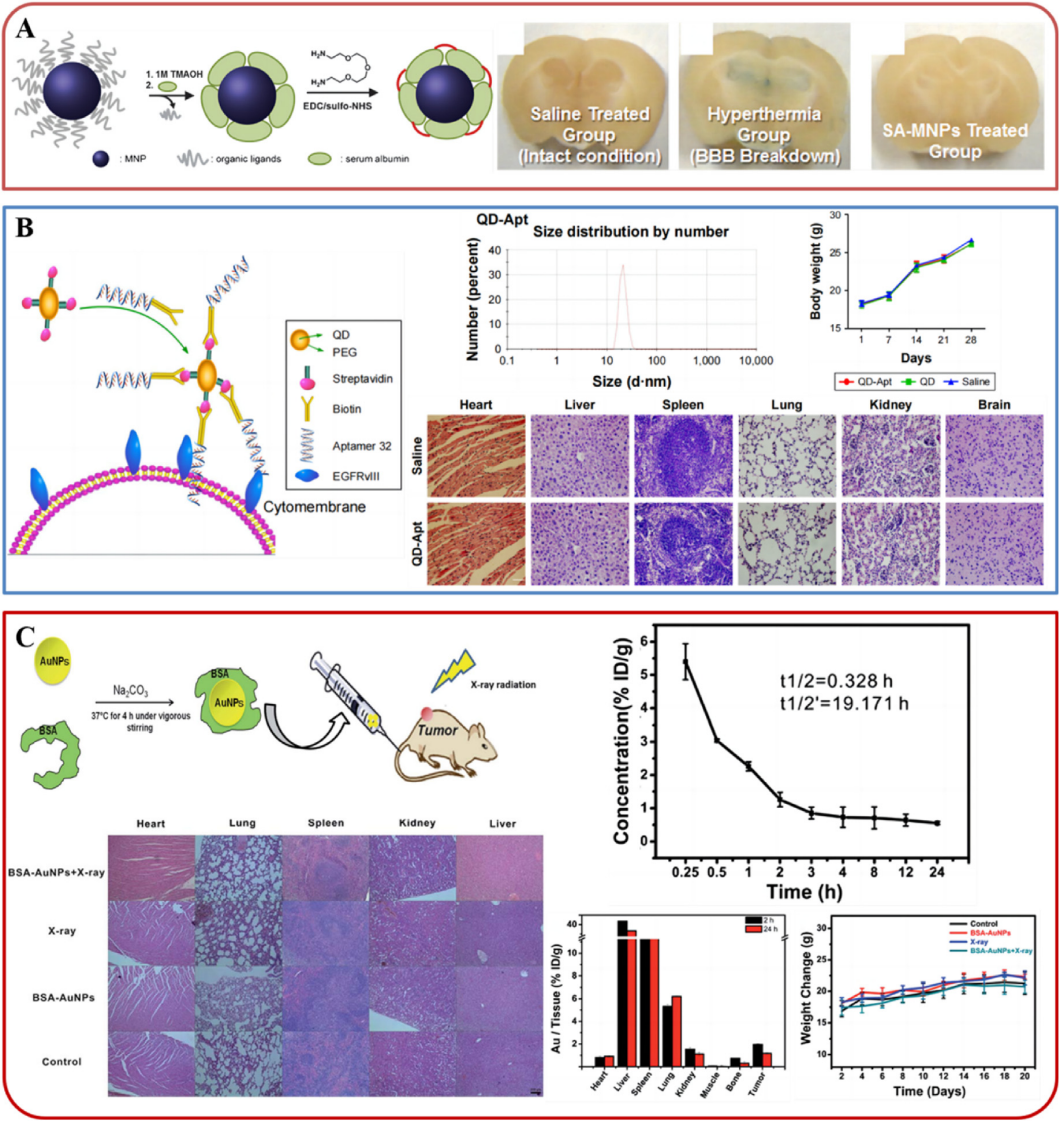
***In vivo* compatibility evaluation of INPs used for glioma treatment.** A. SA-MNPs; B. QD-Apt; C. BSA-AuNPs; Panel A is adapted with permission from Ref. [102], copyright 2012 Chemical Communications. Panel B is adapted with permission from Ref. [75], copyright 2017 International Journal of Nanomedicine. Panel C is adapted with permission from Ref. [106], copyright 2015 RSC Advances.

Firstly, in terms of evaluating the effects of INPs on neurotoxicity, Cheon et al. [102] synthesized CNS-permeable INPs composed of a magnetic metal ferrite nanoparticle core and a cross-linked serum albumin (SA) surface coating (SA-MNPs; Fig. 7A). To evaluate the effects of SA-MNPs on BBB integrity, the BBB leakage was tested using Evans Blue, which does not penetrate the BBB under normal conditions. No brains from SA-MNP-treated animals showed any blue colour, indicating that BBB integrity is not influenced by SA-MNPs. This also confirms their biocompatibility with the BBB structure, which indicates that there are prospects to apply SA-MNPs in nervous system disease treatment.

Immunohistochemical methods such as haemoxylinoglobulin (HE) are generally used to evaluate damage by INPs to major organs *in vivo*. For example, Cheng et al. [75] constructed a novel quantum dot (QD)-labelled aptamer (QD-Apt) nanoprobe by coupling aptamer 32 (A32) to the QD surface (Fig. 7B). This increases the ability to target glioma cells. A32 is a single-stranded DNA capable of binding to the EGFRvIII, which is especially distributed on the surface of glioma cells. The toxicity of QD-Apt was examined *in vivo* through tail vein injection of 5 ​nM QD-Apt in male C57BL/6 mice. The main organs were subjected to histological analysis (HE staining) after treatment. No obvious tissue damage, necrosis or inflammation was observed in the brain or other main organs. The results indicate that the safe doses of Cd-based fluorescent probes for the diagnosis and treatment of glioma are 100 ​nM *in vitro* and 5 ​nM *in vivo*. At a safe concentration, the QD-Apt nanoprobe provides a novel and effective strategy for obtaining preoperative diagnosis, intraoperative resection and postoperative examination of gliomas.

The distribution and excretion of INPs in the main organs of the body are vital indicators of *in vivo* toxicity. Generally, the contents of the main inorganic elements in the blood and major organs are detected and analyzed by mass spectrum, including inductively coupled plasma optical emission spectrometry (ICP-OES) [103], ICP atomic emission spectrometry (ICP-AES) [104] or ICP mass spectrometry (ICP-MS) [105]. Tu's group [106] prepared BSA-capped Au NPs (BSA-AuNPs) as an efficient sensitizer for glioma radiation therapy (Fig. 7C). ICP-AES was used to investigate the level of Au in the blood and organs of mice after intravenous injection of 1.3 ​mg/mL BSA-AuNPs. Time-dependent analysis of blood Au levels first showed that rapid clearance of BSA-AuNPs occurred within the first 2 ​h. Then, the mice were continuously irradiated for 2 ​h and 24 ​h after intravenous injection of BSA-AuNPs. The ICP analysis of the various organs showed that there was no significant difference in Au levels between 2 ​h and 24 ​h. Finally, the long-term (20 day) biocompatibility of BSA-AuNPs was also evaluated by recording the weight of mice and performing HE staining of their main organs, which indicated no obvious damage. In brief, these results confirm that BSA-AuNPs have satisfactory biocompatibility as promising sensitizer candidates for glioma radiation therapy.

*In vitro* and *in vivo* studies have found numerous inorganic nanomedicines with great application prospects for glioma treatment. However, there is still very limited research work explored the bio-safety of INPs at pathological level, such as BBB integrity, brain structure damage and inflammation; as well as the long-term toxicity to brain functions, including cognitive function, memory, behaviour, etc. However, with the further development of nanomedicine and the need for transformation, brain damage should be evaluated more deeply before the use of nanomedicines.

## 5 Diagnostic strategies for glioma based on INPs

Due to the infiltrative growth characteristics of gliomas, they have no clear boundary with normal tissue; thus, gliomas cannot be cured by surgery alone. This means that we urgently need to develop clear imaging tools and contrast agents to surmount the current treatments and diagnosis limitations. Inorganic nanomaterials are excellent PSs and contrast agents and can realize early tumor diagnosis, image mediation and real-time treatment monitoring. Hence, they have been widely used in the biomedical imaging and diagnosis of gliomas. A variety of physical, chemical and biological diagnostic methods, including MRI, computed tomography (CT), positron emission tomography (PET), PAI and fluorescence imaging, have substantially benefited the diagnosis of tumor outcomes.

### 5.1 Magnetic resonance imaging

**Fig. 8:**
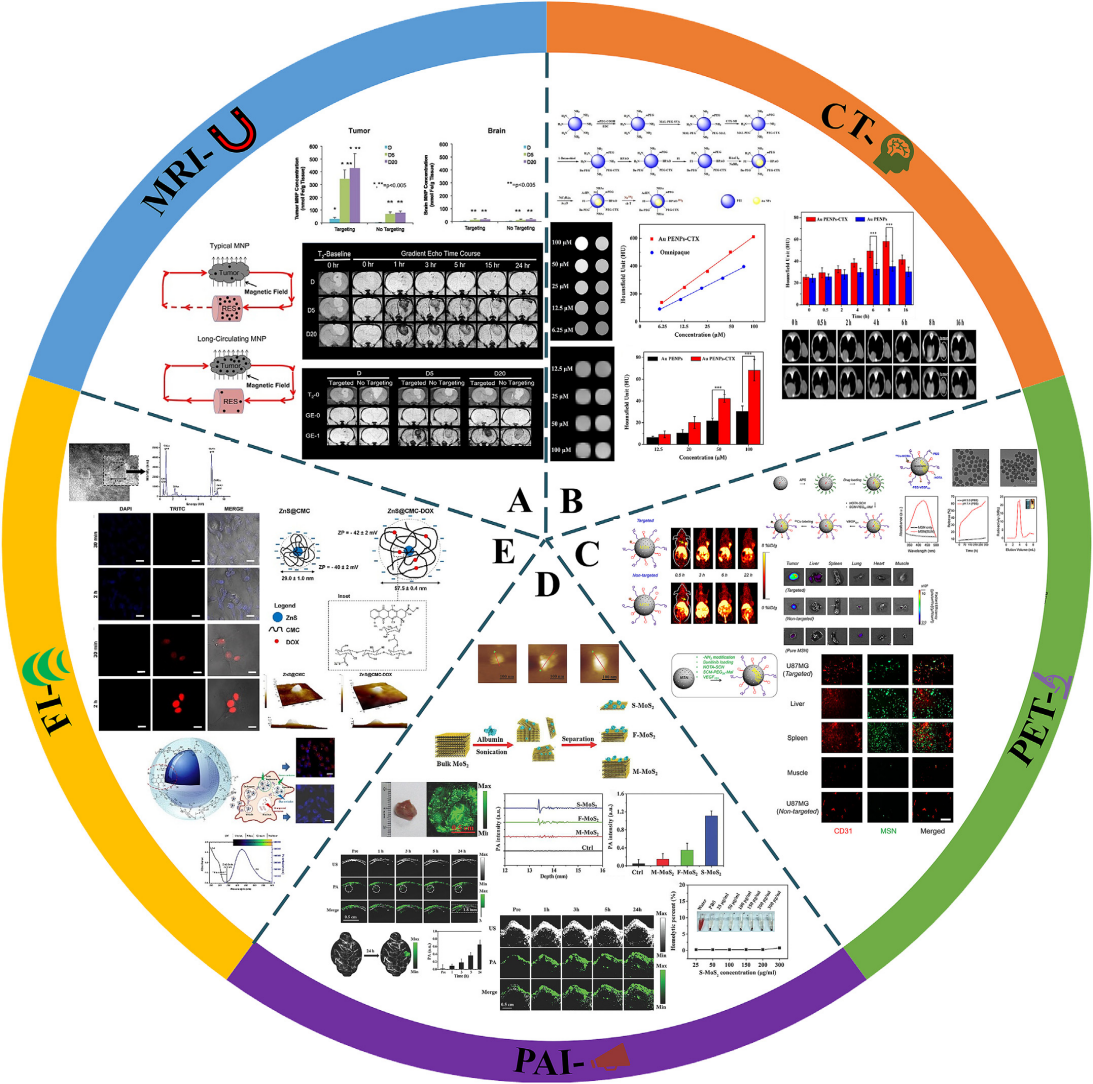
**Diagnostic strategies for glioma based on INPs.** A. PEG-IONPs; B. Au PENPs; C. MSNs; D. S–MoS_2_ Nanosheets; E. PEGNIO/Qds/MIONS/Tf; Panel A is adapted with permission from Ref. [118], copyright 2011 Elsevier. Panel B is adapted with permission from Ref. [72], copyright 2019 Springer Nature. Panel C is adapted with permission from Ref. [139], copyright 2014 American Chemical Society. Panel D is adapted with permission from Ref. [149], copyright 2016 Wiley-VCH. Panel E is adapted with permission from Ref. [162], copyright 2019 Elsevier.

MRI is a universal standard for clinical diagnosis and preoperative localization of gliomas. Because of its high spatiotemporal resolution (0.2–1 ​mm), high sensitivity (10^−3^–10^−5^ ​mol/L), non-invasiveness, and multi-directional, multi-sequential and multi-parametric imaging capabilities, it is particularly suitable for the diagnosis of intracranial diseases [107]. The main obstacles to glioma diagnosis are the unclear boundaries of malignant gliomas and the efficiency of the magnetic material in penetrating the BBB and targeting glioma cells [65,108,109]. The aggressive growth of gliomas often results in enlarged blood vessels and abnormal leakage of endothelial cells, as well as impaired lymphatic drainage [110]. However, based on their high-EPR effects, INPs coupled with active targeting molecules can greatly improve the effect of nuclear magnetic imaging of glioma lesions [108]. Among the nanomaterials used as magnetic sensitizers, IONPs have been extensively studied because of their high longitudinal and transverse relaxation values, superior imaging performance, and low cytotoxicity [111]. Numerous works have confirmed that iron oxide nanoparticles have promising applications as MRI T1/T2/T2∗ contrast agents [[112], [113], [114]]. IONPs can enhance the cellular internalization of tumor cells and active phagocytes to improve the visualization of intracranial tumors within healthy brain tissue and slow down the clearance of tumor sites [115] so as to better depict glioma boundaries [116]. Yang et al. [117] reported that PEG-coated IONPs have a long circulating lifetime in the brain of 9 ​L glioma rats, and their enhanced ability to target glioma tissues was confirmed by MRI and histological analysis (Fig. 8A). In Yang and his colleagues’ work, PEG-coated IONPs exhibited high colloidal stability, which improved magnetic capture and retention time at the tumor site. In subsequent biodistribution studies, they also demonstrated that PEG-MNPs selectively enhance brain tumor targeting by improving the circulation lifetime of MNPs [118].

In addition to the iron mentioned above, Marc-Andre’ Fortin et al. [119] developed ultra-small gadolinium oxide nanoparticles to label GL-261 GBM polymorphic cells and subsequently localize them *in vivo* for tumor visualization by using MRI. A relaxometric study showed that the relaxation ratio (r2/r1) of DEG-US-Gd_2_O_3_ is suitable for T1-weighted imaging [120,121]. The designed nanostructure contains about 400 Gd ions per 3-nm diameter nanoparticle, producing a signal increase at least 200 times more specific per unit of contrast than that of Gd-DTPA chelates. Conjugates such as DTPA, DOTA and DTPA-BMEA are used to coat Gd, since direct overexposure to gadolinium is toxic [122]. Not only that, the tumor retention time of this material is longer than that of Gd-DTPA. Compared with the Gd-DTPA used by de Stasio [123], Fortin's procedure required only 0.6 ​mg of Gd/mL DEG-US-Gd_2_O_3_ and 8 ​h incubation to reach the higher residual concentration of Gd, which was at least six times that of Gd-DTPA. During *in vivo* experiments, they used the chorioallantoic membrane of chick embryos as a tumor growth model and studied it using a 1.5 ​T clinical MRI scanner. At 14, 38, and 84 ​h after inoculation, the labelled cancer cells were clearly seen as they showed obvious contrast with the unlabelled normal tissue cells. GL-261 glioma cells appeared bright on the T1-weighted MRI images, indicating that the very-high-concentration Gd was effectively internalized and retained in the cells. This also laid a foundation for the development and use of Gd-based NMR contrast agents. Other elements commonly used for MRI of glioma include Fe, Au, Mn, Ce, Gd and Dy, which also have high imaging sensitivity and are good choices for use as contrast agents [58,119,124,125].

### 5.2 Computed tomography and positron emission tomography

CT and PET are widely employed modalities for intracranial tumor diagnosis. They can also be used to observe tumor images inside the substantial organs. However, the diagnosis of glioma still lacks sufficient accuracy. This may be related to early neuroinflammation, late radiation necrosis, or tumor recurrence [[126], [127], [128]]. Inorganic nanomaterials, especially magnetic nanoparticles, semiconductor materials and metal elements with high atomic numbers such as Au [72], Ag [129], Yb [130] and Bi [131], are reported to act as enhancers for CT and PET scanning and diagnosis of glioma due to their high X-ray absorption capacity, favourable biocompatibility and excellent optoelectronic properties [132,133].

Zhao et al. [72] designed a multifunctional targeting nanoprobe from Ref. [131]I-labelled CTX-functionalized Au PENPs ([131]I–Au PENPs-CTX). The surface of polyethylenimine (PEI) was modified with 3-(4-hydroxyphenyl) propionic acid-OSu and CTX to entrap AuNPs. CTX, a 36-amino-acid peptide with strong affinity for matrix metalloproteinase-2 (MMP-2) on glioma cells, was used as a targeting ligand and the radionuclide [131]I was used to label CTX [134,135]. *In vitro* experiments conducted on the C6 cells found that, compared with Omnipaque (a small-molecule CT contrast agent used clinically), the CT images of Au PENPs-CTX were brighter. With increases in gold and iodine concentration, the CT values were further enhanced. The X-ray attenuation characteristic of this nanoparticle was stronger than Omnipaque, while its high X-ray attenuation was associated with favourable CT imaging capability, demonstrating its excellent application potential [136]. *In vivo* experiments were consistent with *in vitro* evaluations, and quantitative measurements 8 ​h after injection showed that tumor CT values were 1.7-fold higher in mice treated with Au PENPS-CTX than in those treated with Au PENPs (Fig. 8B). To sum up, AuNPs were used as a sensitizer to link CTX with enhanced glioma targeting. This provides better *in vitro* and *in vivo* imaging performance in C6 glioma mouse models, highlighting the superior application prospects of such nanoparticles in glioma-targeted diagnosis.

PET is a powerful biomedical imaging technology with high sensitivity and quantitative accuracy. It can provide additional information about tumor metabolic processes that can aid in brain tumor differential diagnosis, grading, molecular typing and the differentiation of therapeutic effects from disease recurrence [137,138]. Cai et al. [139] developed a new multifunctional nano-platform, VEGF_121_-conjugated mesoporous silicon dioxide (MSN), as a theranostic nanomedicine for glioma. In the composite nanostructure, VEGF_121_ (as a radiolabelling ligand) can target VEGF and provides early and sensitive lesion detection by PET [140]. MSN was used as a platform for drug loading, facilitating targeted PET detection to enhance the imaging of glioma lesions and achieve a better drug-release effect [64].Cu is also a radio tracer suitable for PET imaging. Cai et al. performed serial whole-body PET on U87 tumor-bearing mice at multiple time points (0.6, 3, 6 and 22 ​h) after injection of targeted and untargeted groups (Fig. 8C). *In vivo* PET imaging showed that tumor accumulation in the targeted MSN group was 3-fold higher than in the non-targeted MSN group, while the specific accumulation occurred in the targeted MSN group as early as 0.5 ​h. The targeted inorganic nanomaterial system described above for radionuclide-based PET imaging provides high sensitivity and the ability to quantitatively analyse whole-body images, which are essential to treatment optimisation.

As different inorganic elements have different physicochemical properties, it is very important to select appropriate materials and construction methods for the preparation of functional nanoplatforms.

### 5.3 Photoacoustic imaging

PAI is a promising non-invasive optical imaging method that uses light pulses to excite exogenous molecular contrast agents to produce ultrasonic waves that can be detected by transducers, generating 3D images of a region of interest [141,142]. The excellent optical properties and surface functionalization features of inorganic nanomaterials have been used to develop exogenous contrast agents for enhanced PAI. Among them, the most commonly used nanomaterials are gold derivatives with favourable SPR absorption mechanisms [141]. Examples include silicon-coated gold NPs for PAI of surface gliomas, hollow gold nanospheres with glioma-targeting properties for PA molecular imaging, and hollow gold nanospheres that generate intense photoacoustic signals and induce effective photothermal ablation of gliomas using hybrid imaging modalities [[143], [144], [145]]. Transition metals also have potential PAI applications. Research shows that molybdenum disulfide (MoS_2_) nanosheets, as a representation, have better near-infrared (NIR) absorbance, stability and biocompatibility than gold and graphene derivatives [[146], [147], [148]]. Based on this property, Zheng et al. designed single-layer (S–MoS_2_) nanosheets and used them to visualize glioma cell boundaries and tumor locations via PAI [149]. A custom-built acoustic-resolution PA microscopy (AR-PAM) system was used to study the PA effect of MoS_2_ nanosheets [150]. Compared with few-layer (F–MoS_2_) and multi-layer (M ​− ​MoS_2_), S–MoS_2_ nanosheets produced longer amplitudes and higher signal intensities. This is because the elastic modulus of MoS_2_ nanosheets increases with decreases in the number of layers and is related to the PA intensity, suggesting high application capability [[151], [152], [153]]. As shown in Fig. 8D, effective tumor retention and PA contrast sensitivity of S–MoS_2_ were demonstrated in a U87 glioma-cell-bearing mice model. The PA signal at tumor sites increased with time after intravenous injection of S–MoS_2_. Moreover, *in vitro* PAI after tumor resection also confirmed that the PA signal at 24 ​h after injection came from tumor tissue. Quantitative experiments showed that, compared with the endogenous PA signal before injection, the signal intensity increased about 54-fold 24 ​h after injection of S–MoS_2_. Overall, S–MoS_2_ nanosheets are a potential contrast agent for highly sensitive PAI of subcutaneous and orthotopic brain tumors in mice. Other inorganic nanomaterials, such as bismuth, cuprum, as well as transition metal-based oxides, all have PAI properties [[154], [155], [156], [157]]. However, there are few reports on their application to glioma medicine and further research is expected.

### 5.4 Fluorescence imaging

Some inorganic QDs with fluorescence imaging properties have promising prospects in the diagnosis and treatment of glioma by fluorescence imaging. Because of the infiltrative nature of gliomas, it is difficult to minimize the number of cancer cells and preserve normal brain tissue intraoperatively. Fluorescence imaging materials enable lesions to fluoresce, helping the surgeon to detect their edges and operate on them [158]. However, current reported fluorescein materials, such as 5-aminoketoglutarate and sodium fluorescein, suffer from low quantum fluorescence rates and poor photostability, which limit their clinical applications. Semiconductor QDs, usually composed of CdSe cores and ZnS shells [159], are widely studied in glioma fluorescence imaging [160,161]. According to this property, Mansur et al. prepared ZnS@CMC nanohybrids for GBM fluorescence imaging. In this work, zinc fluorescent quantum dots (ZnS-Qds; a fluorescent nanoprobe) were chemically modified by carboxymethylcellulose (CMC, a water-soluble capping ligand and bio-functional layer) and coupled with the anticancer drug adriamycin (DOX) to modify drug release. This provided a dual function of bio-imaging and killing of U87 glioma cells [162]. The results show that blue light released by QDs was concentrated in the cytoplasm 2 ​h after injection, while DOX-related intrinsic fluorescence was mainly concentrated in the nucleus (Fig. 8E). Likewise, Zhang et al. reported the use of high-quality CuInS_2_/ZnS QDs—with CuInS_2_ as the core and ZnS as the shell—for CD133^+^ glioma fluorescence imaging. The anti-CD133 monoclonal antibody pQDs-CD133 ​mAb was attached to QDs for tumor cell targeting to achieve precise targeted fluorescence imaging of glioma stem cells [160]. In short, the combination of appropriate targeting ligands with fluorescent nanoparticles has promising application prospects in the accurate fluorescence imaging of gliomas.

Various imaging methods for glioma diagnosis using nanoprobes have advantages and disadvantages. MRI is a non-invasive technique that can demonstrate the macroscopic structure of a tumor without causing X-ray damage. This makes it an ideal imaging test that avoids the damage associated with other tests. However, it has some inherent problems, such as poor spatial resolution and long acquisition and scan processing times. On the other hand, CT imaging has high spatial resolution but is not ideal for assessing brain tumors due to its poor soft tissue contrast. PET imaging is highly sensitive but requires a radioactive tracer, which increases the risk of cancer in humans. As a hybrid imaging modality, PAI combines the high contrast and spectral-based specificity of optical imaging with the high penetration depth of ultrasound imaging. This makes it ideal for deep tissue resolution and provides multi-scale, multi-dimensional photoacoustic image information. However, it does not enable better functional imaging and has lower image contrast. Fluorescence imaging has high detection sensitivity, an extremely wide range of applications, strong signal intensity, and the ability to label samples with multiple colors. However, it has the disadvantage of limited penetration and low spatial resolution.

Nanoscale platforms have now been developed to assist in the identification and diagnosis of tumors in clinical research. The Food and Drug Administration (FDA)-approved 30 ​nm MNPs have been used to predict the co-localization of therapeutic NPs with tumors and to quantify MNPs levels using the amount of change in the mean T2 profile [163]. Gadolinium-based contrast agents and other low molecular weight gadolinium chelates, such as Gd-DTPA and Gd-DOPA, have also been used in clinical practice [164,165]. In addition, nanoprobes can be tracked by linking peptides and antibodies that interact with glioma-specific molecules. Nanoprobe tracers that are capable of specifically binding to integrin α_v_β_3_, vascular endothelial growth factor receptor, or IDH-mutations (glioma-specific molecules) have been successfully studied in clinical glioma patients in preliminary studies, paving the way for more nanoprobes to enter the clinic [166]. Overall, the development of nanoscale platforms and the use of imaging modalities can aid in the identification and diagnosis of glioma in clinical research. However, further efforts are needed for the full clinical translation of INPs-based imaging for glioma diagnosis. It is worth noting that INPs-based systems, due to the convenience of nanoscale platform preparation, the ability of different inorganic elements to be doped, the advantages of surface modification, and the addition of biological ligands, can simultaneously obtain multi-modality imaging technologies. The complementary advantages between different imaging modalities can provide more accurate information for the diagnosis of glioma, as detailed in 6.3.1.

## 6 Therapeutic strategies for glioma based on INPs

In clinical practice, gliomas are mainly treated by surgical resection. For unresectable gliomas and postoperative adjuvant treatment, chemotherapy, radiotherapy and gene therapy are mainly adopted. Traditional therapies involving INPs have been proven to enhance therapeutic targeting as well as therapeutic efficacy. On the other hand, the development of INPs has also created new strategies for glioma therapy. The next section introduces traditional methods that are enhanced by INPs, new glioma treatment strategies guided by INPs, and multi-functional nanoplatforms for guided synergistic diagnosis or treatment.

### 6.1 INPs-enhanced strategies in conventional glioma therapy

INPs can cross the BBB and have excellent targeting properties after connecting with targeting groups, which can greatly improve traditional glioma treatment methods. For example, chemotherapy and gene therapy can be effectively carried out by encapsulating chemotherapeutic drugs or gene interference sequences, while INPs can also be used as radiosensitizing agents to improve the efficacy of radiotherapy.

#### 6.1.1 INPs-enhanced chemotherapy

Chemotherapy is still one of the standard treatments applied after maximal safe surgical resection. Despite some breakthroughs [167], glioma therapeutic efficacy remains fairly poor due to inadequate medication at lesion sites. INPs can be employed as drug carriers for glioma chemotherapeutics, enabling more precise and superior therapeutic effects. This is because nanomedicine can be used to transport unsuitable molecules into the brain by binding them or wrapping them in an appropriate vehicle. Therefore, chemotherapy mediated by nanomaterials can provide targeted drug delivery that overcomes the shortcomings of traditional chemotherapy. The combination of chemotherapy medications with INPs [59,124] that can penetrate the BBB can increase biocompatibility while maintaining or promoting the efficacy of chemotherapeutic drugs. Common targeting molecules that can help cross the BBB are shown in Table 2. There are many drugs that can be used for nanomaterial delivery, such as PTX [168], tetrandrinev [169], oleanolic acid [170], DOX [171], CDDP [172], methotrexate [173] and Cur [174]. Among these, DOX—a typical chemotherapeutic agent—is an anthracycline with broad-spectrum anticancer activity that is widely employed in clinical use, including glioma treatment [175,176]. Some of the many carriers suitable for DOX, and their effects, are described next.

**Fig. 9:**
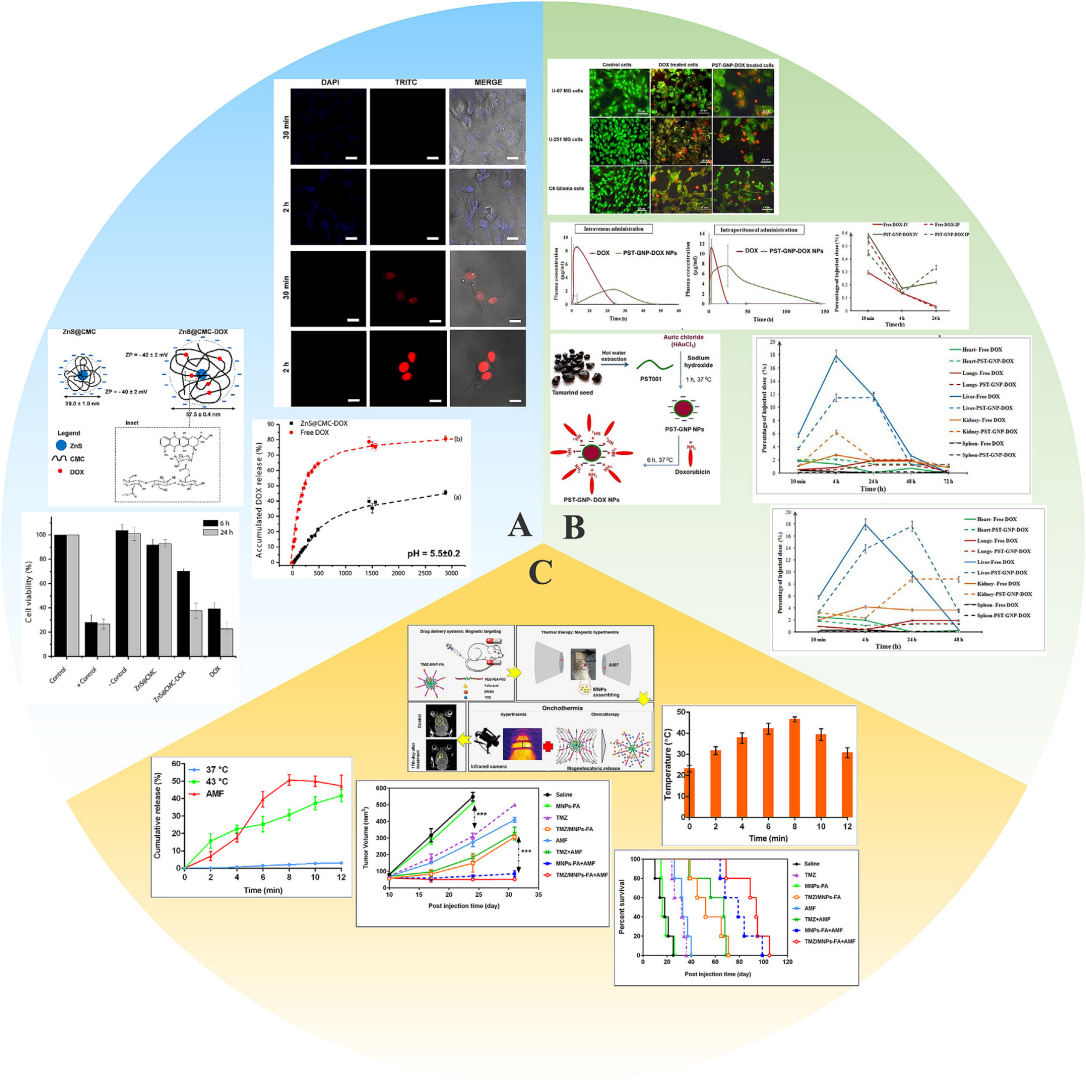
Applications of INPs in glioma chemotherapy. A. ZnS@CMC-DOX; B. PST-GNP-DOX NPs; C. TMZ/MNPs-FA. Panel A is adapted with permission from Ref. [162], copyright 2019 Colloids and Surfaces B: Biointerfaces. Panel B is adapted with permission from Ref. [179], copyright 2019 Nanomaterials. Panel C is adapted with permission from Ref. [182], copyright 2021Nanomedicine.

Mansurand and colleagues developed innovative supramolecular complexes (ZnS@CMC-DOX) composed of an inorganic ZnS quantum dot core (ZnS-QD) biofunctionalized with a CMC polymer shell (ZnS@CMC) and conjugated with the DOX anticancer drug (Fig. 9A) [162]. Non-toxic binary ZnS semiconductor QDs have been designed to construct drug delivery systems [177]. An aqueous medium at pH ​= ​5.5 ​± ​0.2 (CE ​> ​99%) was selected to simulate the acidic microenvironment of cancer cells [178]. ZnS@CMC-DOX nanoconjugates exhibited a drug release rate of approximately 45% after incubation for 2 days, while the ‘free DOX’ release rate exceeded 80%. As a result, the drug release rate was slower after loading into the nanocomposites, which could increase the duration and action time of the drug and avoid the toxic side effects caused by rapid release. Other evidence illustrates that ZnS@CMC nanoconjugates without an anticancer drug load were not cytotoxic to normal cells and brain cancer cells. After loading with DOX, ZnS@CMC-DOX hybrid complexes retained a lower DOX cytotoxicity than free DOX, which was reflected in the following results. The cell viability responses of normal and tumor cells exceeded 70% after incubation of ZnS@CMC-DOX for 6 ​h. Cell viability was further reduced to 38% with the extension of culture time (24 ​h). Meanwhile, the cell viability of the free DOX group decreased to about 35–40% after 6 ​h. Consequently, the above studies prove that the ZnS@CMC-DOX nanocarrier can decrease the cytotoxicity in the short term and improve the sustained release effect of DOX. This reduces damage to normal tissue but does not affect the simultaneous cytokilling efficacy of DOX, making it an ideal carrier for drug delivery.

GNPs are an attractive drug carrier candidate due to their reasonably low cytotoxicity, adjustable surface properties and stability under *in vivo* conditions. Komeri et al. [179] synthesized GNPs capped with galactoxyloglucan PST001 isolated from the seed kernels of ripened *Tamarindus indica* fruit. This was employed as a DOX nanocarrier and named PST-GNP-DOX NPs (Fig. 9B). PST001 has innate antitumor and immune-potentiating functions and was used as a GNPs reducing and stabilizing agent without introducing any environmental toxicity or biohazard [180]. The efficacy of PST-GNP-DOX NPs to glioma cells was confirmed using apoptotic staining. It is worth highlighting that the cell density of glioma cells treated with PST-GNP-DOX NPs was significantly lower than in cells treated with free DOX, especially at low doses. More importantly, PST-GNP-DOX NPs also had a longer mean residence time in plasma after intravenous and intraperitoneal administration. Meanwhile, PST-GNP-DOX NPs show less accumulation in the heart, lungs and spleen and a more uniform distribution in the brain, which suggests that PST-GNP-DOX NPs could escape from the RES. Neutral nanoparticles with an average diameter of 100 ​nm have been found to be able to evade the RES and mononuclear phagocyte system [181]. PST-GNP-DOX NPs might successfully escape from the RES when they have a mean hydrodynamic diameter of 133 ​nm and a surface charge that is nearly neutral in this circumstance. Besides, PST-GNP-DOX can greatly inhibit the growth of DOX-resistant U87 tumor cells compared with free DOX. Not only that, PST-GNP-DOX NPs also decreased the expression of BCRP efflux protein, which was overexpressed in drug-resistant tumor cells. At the same time, PST-GNP-DOX NPs decreased the expression of NF-κB associated with tumor progression, which may explain the effect of PST-GNP-DOX NPs to overcome drug-resistant gliomas. However, the specific molecular mechanism has not been explored. In brief, PST-GNP-DOX NPs are promising candidates for delivering DOX to glioma tumors.

Afzalipour et al. [182] developed TMZ-loaded magnetic nanoparticles conjugated with folic acid (TMZ/MNPs-FA) to create thermosensitive magnetic NPs (Fig. 9C). To find out how MNPs-FA affected drug release under magnetic and thermal circumstances, they first demonstrated that it could slow down the TMZ release rate compared with free TMZ [183]. The drug release rate increased with increasing temperature from 37 ​°C to 43 ​°C. Within the first 8 ​min of exposure to an alternating magnetic field (AMF), the drug release rate rose dramatically to 52.1 ​± ​2.3% but fell sharply once the AMF was turned off. In the absence of AMF, the release rates were 30% at 43 ​°C and <2% at 37 ​°C. Nanoparticles exposed to a magnetic field generate heat via Neal relaxation phenomenon [184]. Thus, MNPs-FA are heat-sensitive, which can regulate drug release. The results above show that TMZ/MNPs-FA nanoparticles are relatively stable at physiological temperatures (37 ​°C), while under the influence of AMF, the heat generated increases the drug release rate by affecting the polymer packing. This research extensively studied the biomedical application prospects of MNPs-FA in glioma treatment. MNPs-FA demonstrate potential for loading TMZ and transporting it across the BBB, while also showing excellent potential for remote-controlled drug release through heat induction.

Overall, INPs-based delivery carriers were proven that can enhance glioma chemotherapeutic efficacy by improving both the BBB-penetration efficiency and glioma-targeting ability. In addition, some INPs-base carriers can also control the release rates of chemotherapeutic drugs to prolong their action time. However, the long-term biological toxicity of various nano-delivery carriers, as well as the specific mechanisms of clearance and metabolism from the brain are worthy of further investigation.

#### 6.1.2 Radiotherapy

Radiation therapy is a major strategy and common adjunct in the treatment of GBM. One of the major challenges in radiotherapy is minimizing radiation damage to healthy tissue without sacrificing the therapeutic effect on tumor cells. Clinical and experimental studies have revealed many treatment failures and unsatisfactory outcomes due to the radiation resistance of GBM [185]. The introduction of nanomedicine, such as by utilizing INPs as a radiosensitizer, offers an opportunity to enhance the therapeutic index of radiation therapy [186]. In addition, elevated concentrations of INPs in cancer cells might result in substantial accumulation of radiation energy at tumor regions compared with surrounding healthy tissue [187].

**Fig. 10:**
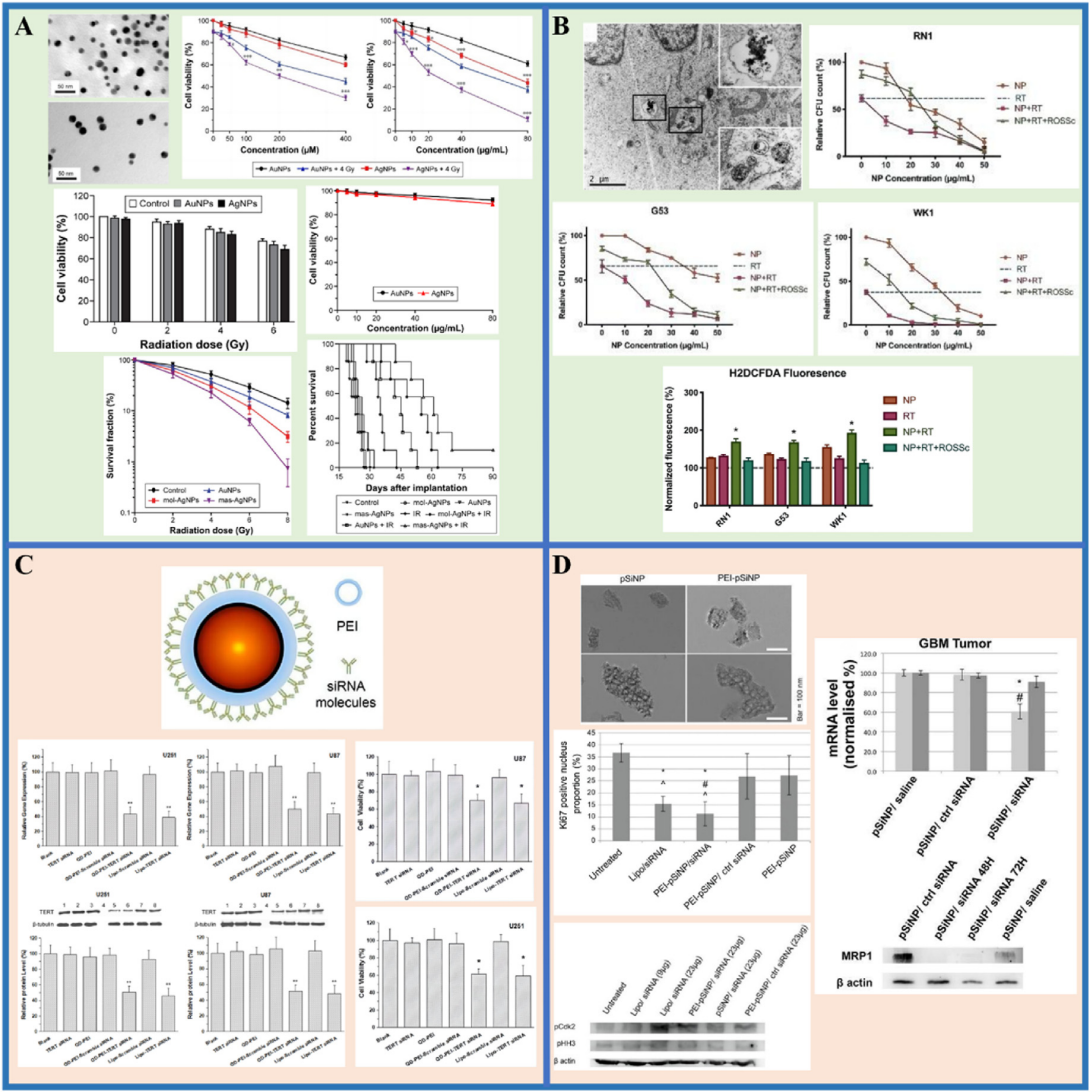
**INPs-enhanced strategies in conventional glioma therapy.** (A–B). The application in radiotherapy; A. Ag/AuNPs; B. La_2_O_3_ NPs. (C–D). The application in gene therapy: C. QD-PEI; D. pSiNPs. Panel A is adapted with permission from Ref. [188], copyright 2016 International Journal of Nanomedicine. Panel B is adapted with permission from Ref. [190], copyright 2020 Scientific Reports. Panel C is adapted with permission from Ref. [194], copyright 2017 Frontiers in Pharmacology. Panel D is adapted with permission from Ref. [196], copyright 2018 Journal of Nanobiotechnology.

Liu et al. [188] compared the radiation-sensitizing effectiveness of AuNPs and AgNPs on glioma (Fig. 10A). The effects of AuNPs and AgNPs in combination with radiation on colony formation by U251 ​cells showed that the survival rates of the cells decreased sharply with increasing doses of 6 ​MV X-rays. The colony-forming assay is the gold standard for detecting radiosensitivity [189]. Notably, AgNPs showed the highest radiosensitizing activity, followed by AuNPs. This might be on account of AgNPs having stronger proapoptotic activity and upregulated autophagy levels. The results also showed that treatment of INPs with or without radiation therapy had little effect on noncancerous cell lines; thus, the prepared nanomaterial-mediated radiosensitization was tumor cell-specific. Next, radiation therapy was performed after the injection of AgNPs or AuNPs intratumorally in a glioma mice model. Kaplan-Meier survival plots confirmed the *in vivo* anticancer effects of AgNPs. Clearly, this work demonstrates the application prospects of AgNPs as highly effective nano-radiosensitizers for the treatment of glioma.

Lu and colleagues [190] investigated the augmented radiation effects of lanthanum oxide (La_2_O_3_) NPs on glioma via cellular ROS mechanisms (Fig. 10B). Due to its special chemical characteristics, lanthanum is cytotoxic to cancer cells and is able to enhance existing anti-cancer therapies. After intravenous injection, La_2_O_3_ NPs can reach the brain and target GBM cells through endocytosis, then dissociate to produce cytotoxicity, enhancing the therapeutic effect of radiation therapy. Radiation therapy with La_2_O_3_ NPs significantly decreased the amounts of colony-forming units (CFUs) and increased the production of ROS in GBM patient-derived cell lines (RN1, G53 and WK1). There was evidence that the amount of CFUs recovered after pre-treatment with a ROS scavenging agent (ROSSc) for 1 ​h. Notably, La_2_O_3_ NPs were associated with markers of ROS-dependent endogenous and exogenous apoptotic pathways, as well as involving direct DNA damage and autophagy pathways. These may be the mechanisms by which La_2_O_3_ NPs sensitize GBM to radiotherapy. These findings imply that cytotoxic La_2_O_3_ NPs can sensitize GBM cells to radiation therapy.

The acceptable intensity of radiation for an organism is limited. It is important to note that all of the mentioned INPs have achieved a radiotherapy-enhanced effect, thereby reducing the radiation hazard. Studies have reported that radiation therapy can cause irreversible CNS damage, so radiation therapy still needs to be chosen carefully in clinical practice. Therefore, safer and more effective radiosensitizers need to be urgently developed and INPs will be one of the options.

#### 6.1.3 Gene therapy

Gene therapy has attracted extensive attention over the past few years due to its novel and effective cancer treatment. Among gene therapy techniques, gene silencing based on RNA interference (RNAi) has emerged as a potential strategy for biological therapy [191]. In addition to therapeutic targeting, another crucial factor for successful gene therapy is obtaining an ideal delivery vehicle with safe and high-efficiency gene knockdown. Since small interfering RNA (siRNA) molecules have difficulty traversing cell membranes and are susceptible to serum nucleases during blood circulation, they can be encapsulated in nanocarriers. This obtains excellent biocompatibility and easy intracellular uptake, enabling efficient knockdown of oncogenes or other related genes *in vivo* [192,193]. In recent years, the application of INPs greatly improve the effectiveness of glioma gene therapy via enhanced tumor targeting (connecting targeted groups) and BBB permeability.

Yong's group [194] fabricated cadmium sulphoselenide/zinc sulfide quantum dot (CdSSe/ZnS QD)-based nanocarriers conjugated with PEI to form a stable nanocomplex (QD-PEI). This was used to load siRNA specifically to GBM cells by targeting human telomerase reverse transcriptase (TERT). TERT is the catalytic protein subunit of telomerase, which is crucial for maintaining telomerase activity in cancer cells [195]. The excellent optical properties of QDs enable real-time monitoring of the siRNA delivery process. The expression of TERT mRNA and protein in human glioma cell lines (U87/U251) transfected with QD-PEI-TERT siRNA nanocomplex was found to be significantly inhibited, as shown in Fig. 10C. This demonstrates the efficiency of gene silencing and provides a stable platform for gene therapy applications. Next, the author investigated the targeted gene therapy ability of a QD-PEI-TERT siRNA nanocomplex. Comparing with controls, tumor cells were considerably fewer in a QD-PEI-TERT siRNA treatment group. Hence, the QD-PEI developed in this study can successfully deliver siRNA interference sequences, which is anticipated to be a candidate delivery vector for glioma gene therapy.

Voelcker et al. [196] established porous silicon NPs with a PEI capping (PEI-pSiNP/siRNA), which enables high-capacity loading and delivery of siRNA for GBM gene therapy. Overexpression of multidrug resistance-associated protein 1 (MRP1) plays an important role in chemotherapy resistance in GBM, leading to lethal consequences [197]. As shown in Fig. 10D, the INPs successfully downregulated MRP1 expression by 30%. Compared with untreated controls, PEI-pSiNP/siRNA treatment significantly depressed the expression level of Ki67 (a marker of cell proliferation) [198] in U87 ​cells, which might lead to the inhibition of GBM proliferation. Furthermore, their results indicated that *in vitro* observations are translatable *in vivo*. In summary, efficient MRP1-siRNA delivery using PEI-pSiNP/siRNA might achieve a dual-GBM therapeutic role of chemotherapeutic sensitivity and tumor suppression.

As a safe and efficient gene knockout vector, INPs can be used for adjuvant therapy of glioma in many aspects, such as the knockout of the mentioned above genes that maintain telomerase activity of cancer cells or play an important role in GBM chemotherapy resistance. Therefore, such targeted gene therapy to promote glioma treatment will be an interesting endeavor.

### 6.2 Novel glioma therapeutic strategies guided by INPs

INPs have provided new treatment methods for glioma due to their unique properties. On the one hand, INPs can be used as immunogenic cell death (ICD) inducers to improve the efficacy of immunotherapy. On the other hand, they can be used as PSs or photothermal agents (PTAs) for the PDT or PTT of gliomas. It is also possible to treat gliomas with magnetic hyperthermia in the presence of AMF. However, these novel therapies are still mainly the subject of research and have not been clinically applied.

#### 6.2.1 Phototherapy

Tumor phototherapy has attracted particular attention because it has greater efficacy and less invasiveness than conventional cancer therapies [[199], [200], [201]]. PDT and PTT are the most prevalent phototherapy strategies, both of which are effective in destroying tumor cells by producing ROS or providing selective local heat via laser absorption [109]. PDT is a minimally invasive procedure that combines PSs, a specific wavelength of laser and molecular oxygen, which might be selectively retained by pathological tissue and not normal tissue when administered to patients [202,203]. PDT-mediated tumor death is generally an active death process, occurring mainly by autophagy and apoptosis [204]. The temperature range of PTT is 41–47 ​°C, which can achieve the purpose of tumor ablation by inducing the denaturation of proteins in tumor cells and causing their irreversible death [[205], [206], [207]]. However, due to the complexity of the brain structure, high temperatures are difficult to tolerate. Therefore, many scholars have proposed using low-temperature phototherapy at about 41–43 ​°C for the PTT of gliomas. In addition, if the nanocomposites have a drug-delivery function, the photothermal effect will also lead to drug release. Then, the nanocomposites can be combined with phototherapy to achieve photothermal and chemotherapy-enhanced therapeutic effects and regulate the drug release rate [208].

INPs have become essential materials in the field of glioma phototherapy due to their physical properties, and high physiological stability and biocompatibility. Furthermore, NIR lasers possess high bio-penetration and retention properties [209], while the rapid development of nanotechnology has made it possible to apply NIR-excited lasers in both *in vitro* and *in vivo* therapies. The skull and scalp can severely attenuate laser transmissions so that only a small proportion of the energy reaches the glioma site. Therefore, during glioma phototherapy, INPs excited by long wavelengths (>900 ​nm) are better for PDT/PTT treatment of glioma than traditional PSs/PTAs [210].

**Fig. 11:**
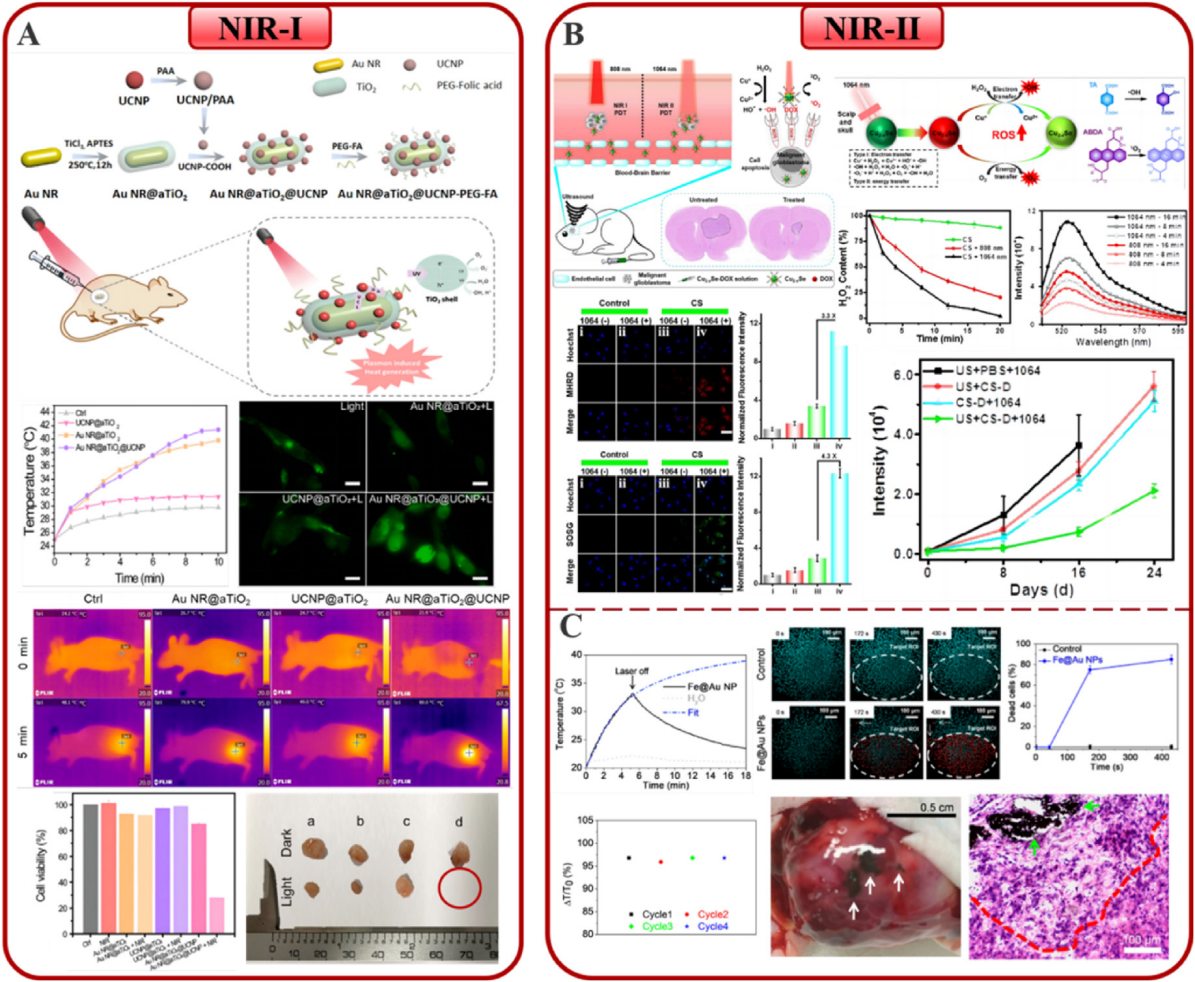
**INPs used in glioma phototherapy.** A. Au NR@aTiO_2_@UCNPs; B. Cu_2-x_Se NPs; C. Fe@Au NPs. Panel A is adapted with permission from Ref. [213], copyright 2021 ACS Applied Materials & Interfaces. Panel B is adapted with permission from Ref. [80], copyright 2019 Nanoscale. Panel C is adapted with permission from Ref. [215], copyright 2021 pharmaceutics.

Currently, various INPs, such as noble metal nanoparticles [77,211,212] and graphene QDs [100] have been employed for NIR laser-mediated PDT/PTT of glioma. Many NIR-I-activated INPs with multiple optical functions can be used to treat gliomas. Kim and colleagues [213] developed anatase titania-coated plasmonic gold nanorods decorated with upconversion nanoparticles (Au NR@aTiO_2_@UCNPs; Fig. 11A). This novel architecture incorporates an anatase titanium dioxide (aTiO_2_) photosensitizer as a spacer and takes advantage of the localized surface plasmon resonance (LSPR) properties of the Au core. A superior ROS-producing ability was found with Au NR@aTiO_2_@UCNPs under laser irradiation (808 ​nm, 2 ​W/cm^2^) compared with controls. Meanwhile, the temperature of Au NR@aTiO_2_ and Au NR@aTiO_2_@UCNPs could reach 42 ​°C after 10 ​min under NIR irradiation. This excellent low-temperature photothermal efficiency is expected to play a key role in assisting glioma PDT. The heating is primarily attributed to the plasmonic effect of the Au NR cores. *In vitro* tumor cell ablation research demonstrated that Au NR@aTiO_2_@UCNPs facilitated a remarkable reduction in cell viability once activated. An *in vivo* anticancer experiment also showed that complete glioma ablation could only be achieved by NIR laser irradiation in the presence of Au NR@aTiO2@UCNPs. Overall, Au NR@aTiO_2_@UCNPs are excellent multifunctional phototherapeutic agents that can provide a valuable complement to laser-triggered deep-tissue glioma PTT/PDT.

Compared with NIR-I lasers (750–900 ​nm), NIR-II region lasers (1000–1700 ​nm) have deeper tissue penetration ability, a higher radiation ceiling and better tissue tolerance. They also have greater potential for application in the phototherapy of brain tumors [214]. However, there are few reports on the treatment of deep-seated gliomas by NIR–II–mediated phototherapy. Gao et al. [80] reported the treatment of *in situ* GBM with imaging-guided NIR-II PDT and chemotherapy using Cu_2-x_Se (CS) NPs (Fig. 11B). These drug-loaded ultra-small theranostic NPs can cross the BBB with the assistance of ultrasound and act as both a photosensitizer and therapeutic agent. After a CS NPs/H_2_O_2_ mixture was irradiated with a 1064 ​nm laser, H_2_O_2_ was efficiently degraded at a rate higher than that attained at 808 ​nm. Meanwhile, stronger ROS fluorescence was also observed under 1064 ​nm irradiation. Subsequently, the authors demonstrated that CS NPs effectively degraded H_2_O_2_ and oxygen within the tumor via electron transfer and energy transfer mechanisms. Next, a low concentration of CS NPs (25 ​μg/mL) was incubated with U87 ​cells and irradiated for 5 ​min to detect intracellular ·OH and ^1^O_2_ radical production. The cells showed intense red (·OH) and green (^1^O_2_ radicals) fluorescence, which was 3.3- and 4.3-fold higher than that of unirradiated CS NPs cells, respectively. To sum up, the advantages of ultra-small Cu_2-x_Se NPs combined with a deeply penetrating 1064 ​nm laser show great potential in NIR-II PDT combined therapy for glioma. Additionally, García-Martín et al. [215] investigated the photothermal properties of gold-coated iron oxide NPs coated with polyvinylpyrrolidone (Fe@Au NPs; Fig. 11C). Fe@Au NPs exhibit an exploitable “photothermal” conversion ability in the NIR-II region due to the existence of LSPR in gold. As expected, under a NIR laser (1064 ​nm, 1.22 ​W/cm^2^), Fe@Au NPs demonstrated a rapid increase in temperature under laser heating and cease after laser shutdown. The heating rate of the NP suspension was 3.3 ​°C/min, which was 10 times that of the control. In addition, during four cycles of NIR irradiation, the maximum temperature remained similar, proving the excellent stability of Fe@Au NPs under NIR irradiation. Notably, the calculated photothermal conversion efficiency was 42.6%. *In vitro* irradiation experiments showed that >90% of the glioma cells were killed in the target region within 7 ​min. Finally, Fe@Au NPs were successfully used for PTT against GBM in a mouse model. These results imply that Fe@Au NPs have the potential to effectively eliminate GBM as thermal-coupling agents *in vitro* and *in vivo*.

Currently, the majority of INPs applied in glioma phototherapy are those that respond to NIR-I, while exploration of phototherapy materials responding to NIR-II with better penetration ability is urgently needed. Moreover, some phototherapy materials used *in vitro* may not have satisfactory anti-glioma effects *in vivo* due to their failure to penetrate the skull under short-wavelength excitation or the high temperatures of PTT, which can result in damage to other brain tissues. Hence, INPs used for phototherapy need to be further optimized.

#### 6.2.2 Immunotherapy

Immunotherapy is mainly used for advanced tumors. Current immunotherapy techniques include immune checkpoint blocking, overwhelming immune tolerance, modified T-cell therapy, and the identification of novel tumor antigens using next-generation sequencing. Passive cancer immunotherapy involves the use of drugs such as lymphocytes, cytokines, and monoclonal antibodies to promote an existing antitumor response. Active cancer immunotherapy, on the other hand, involves stimulating an autoimmune response to invading tumor cells through non-specific immune regulation, vaccination, and explicit targeting of antigen receptors. In fact, only a small proportion of patients benefit from the immunotherapy described above. One of the leading reasons is that tumor cells have a high mutation rate and produce new antigens, resulting in low immunogenicity and poor recognition ability in dendritic cells (DCs), which allows tumor cells to evade the immune response.

In 2022, Vemana [216] outlined NPs-based nanomedicine in targeted delivery of immunotherapeutic medications and immunomodulatory chemicals, and their role in regulating the tumor microenvironment to promote tumor immunotherapy. Several *in vitro* and *in vivo* studies have shown promising effects of NPs in cancer immunotherapy, including significant anti-degradation drug protection, intracellular delivery, regulated and sustained release, and the prevention of multi-drug resistance in various types of NPs. Compared with other immunotherapy, NPs-based immunotherapy can provide durable vaccine efficacy and broad immune responses. Firstly, NPs are the most typical delivery vehicles used to ensure that tumor antigens reach lymph nodes and achieve effective immunotherapy. Secondly, NPs with specific functionalization can induce, inhibit or alter the innate immune system; for example, by inducing cytokine production or by activating downregulation mechanisms or immunosuppressive immune cells [[217], [218], [219], [220]]. In addition to tumor antigens, NPs can efficiently deliver adjuvants to antigen-presenting cells (APCs; e.g., macrophages and DC) located in lymph nodes, thus allowing antigen presentation.

At present, a novel use of NPs in cancer immunotherapy is to promote the maturation of DCs, activate NK cells or T cells, and jointly release immune factors (e.g., TNF-α, IL-6 and IL-1β) to achieve ICD [[221], [222], [223], [224]]. In this part, the ICD therapy for glioma based on INPs was mainly reviewed.

**Fig. 12:**
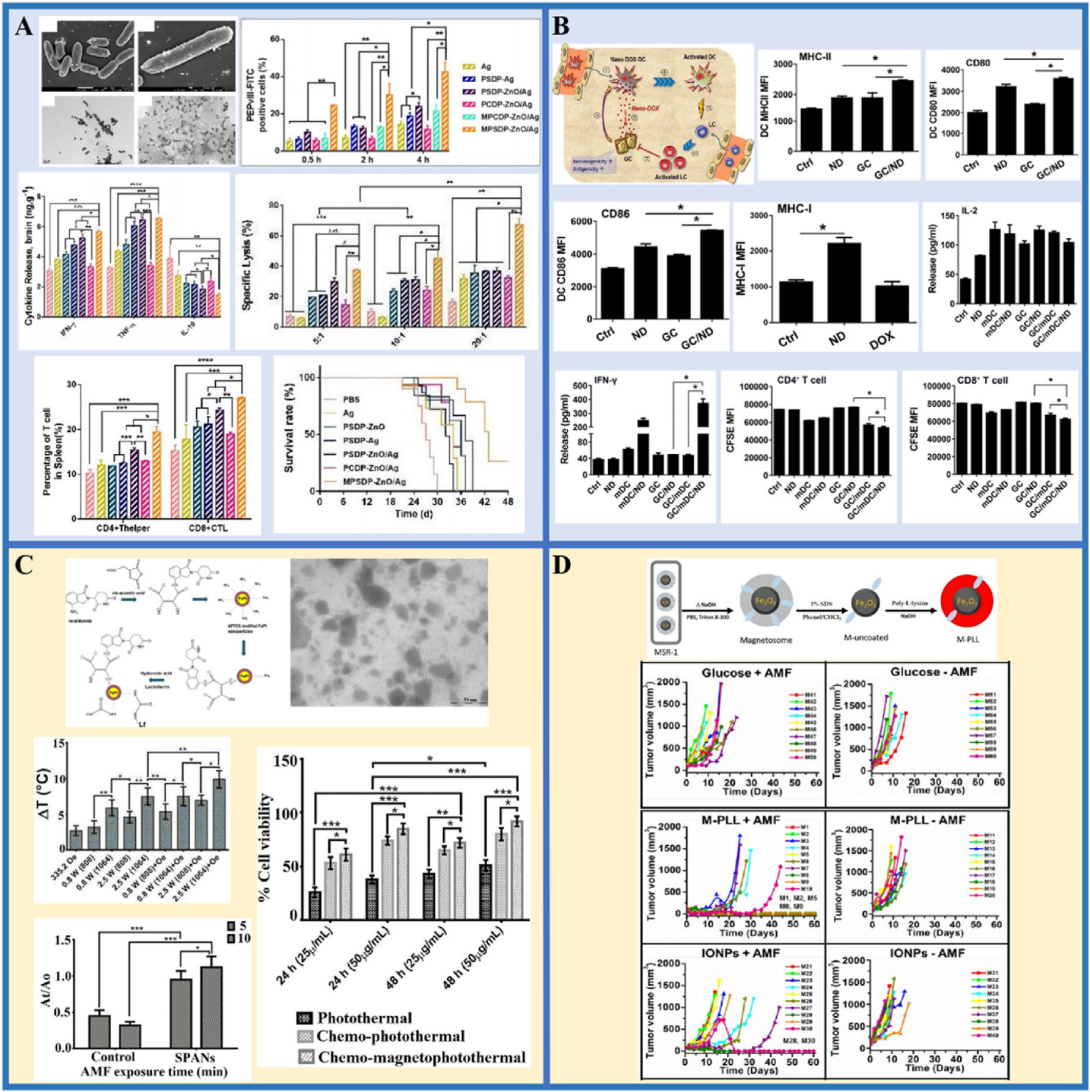
**Novel glioma therapeutic strategies guided by INPs.** (A–B). The application in immunotherapy; A. MPSDP-ZnO/Ag; B. Nano-DOX. (C–D). The application in MHT: C. SPANs; D. IONPs. Panel A is adapted with permission from Ref. [225], copyright 2019 Materials Horizons. Panel B is adapted with permission from Ref. [227], copyright 2018 Biomaterials. Panel C is adapted with permission from Ref. [239], copyright 2018 Materials Science & Engineering C. Panel D is adapted with permission from Ref. [240], copyright 2017 Theranostics.

Zhang and colleagues [225] reported hybrid ‘clusterbomb’ nanovaccines (MPSDP-ZnO/Ag) that can activate the *in vivo* immune response via the lymphatic vessels. MPSDP-ZnO/Ag with high antigen loading and well-defined hybrid nanostructures can trigger a cluster to ‘bomb’ in response to APC for simultaneous release of antigen and adjuvant. Hence, the nanosystem can be applied for the combined immunotherapy of GBM without crossing the BBB. As shown in Fig. 12A, MPSDP-ZnO/Ag can significantly increase the cellular uptake of Ag in DCs and generate EGFRvIII-specific CD8^+^ T cells from vaccinated rats，thus trigger the strongest Ag-specific cytotoxicity. Subsequently, it was also shown that MPSDP-ZnO/Ag might effectively stimulate the proliferation of CD4^+^ T and CD8^+^ T cells in the spleen of tumor-bearing rats, which may be attributed to the high cell-uptake efficiency and intracellular transfer of APC. Furthermore, the expression of anti-inflammatory cytokine IL-10 was significantly reduced, while pro-inflammatory cytokines IFN-γ and TNF-α were significantly increased in the glioma microenvironment after treatment with MPSDP-ZnO/Ag nanovaccine. Notably, the survival rate of the tumor-bearing rats treated with MPSDP-ZnO/Ag was distinctly prolonged. The designed INPs clearly boosted the maturation of DCs and cytokine secretion, which ultimately enhanced immunotherapy against GBM.

In the carbon nanomaterials family, nanodiamonds are a special class of INPs due to their unique physicochemical properties [226]. DOX-polyglycerol-nanodiamond composites (Nano-DOX) [227] can serve as a DOX-based ICD inducer (more potent than free DOX) that can be delivered into GBM via DCs and, subsequently, initiate an anti-GBM immune response (Fig. 12B). The immunogenicity includes an adjuvantability conferred by the release of damage-associated molecular patterns (DAMPs) and antigenicity involved in the production and supply of antigens. The emissions of DAMPs (CRT, ATP, HMGB-1 and HSP90) were significantly higher in Nano-DOX-treated GBM cells (GCs) than in free DOX-treated GCs. The MHC-I upregulation stimulated by Nano-DOX caused more cytoplasmic antigens to be displayed on the GC surface. Moreover, DC must be mature/activated in order to present antigens to the lymphocyte (LC). The authors found that nano-DOX can enhance DC maturation and activation. DC shows a mature morphology and significantly upregulated surface expression of MHC-II, CD80 and CD86, indicative of enhanced antigen presentation. Next, in the presence of nano-DOX, mouse LC (CD4^+^ and CD8^+^) showed marked proliferation when co-cultured with GC and mouse bone marrow-derived DC (mDC), and the release of IFN-γ, a characteristic cytokine of LC activation, was sharply increased. Release of IL-2, another hallmark of LC activation, was increased in all culture environments compared to controls. Lastly, enhanced infiltration and activation of mDC and LC, as well as increased GC apoptosis, were also detected in GBM xenografts treated by nano-DOX. Taken together, the DC-mediated delivery of nano-DOX appears to elicit robust anti-GBM immunity by stimulating immunogenicity in GCs, providing a novel tool for anti-GBM immunotherapy that has great therapeutic potential.

INPs can be passively or actively targeted to gliomas by the EPR effect or ligand surface modification, respectively. Thus, nanotechnology offers a variety of options for efficient delivery of ICD inducers to gliomas at optimal doses due to the development of various construction processes [[228], [229], [230]]. INPs can be used as a local source of ICD induction therapy or to enhance therapy by external energy fields to reduce damage to healthy tissues [231]. INPs with imaging capabilities can be used to monitor their localization and therapeutic effects in real time, while INPs with immunomodulatory effects can be used as adjuvants or immune enhancers [231]. However, based on immunotherapy and nanotechnology, improving the abilities of INPs to cross the BBB and target gliomas, and promoting the clinical translation of glioma immunotherapy, are challenges that remain to be addressed.

#### 6.2.3 Magnetic hyperthermia therapy

Other therapies, such as magnetic hyperthermia therapy (MHT) using MNPs composed of inorganic elements [94,232], have been explored for glioma treatment. In general, MNPs can be exited in the presence of AMF and generate heat through hysteresis loss [233]. The heating capacity depends on the properties of the MNPs and the AMF parameters. MHT was approved as an adjuvant therapy for recurrent GBM in combination with radiotherapy in Europe since 2012 [234]. In clinical trials using MHT to treat GBM patients, an AMF with an intensity of 18 ​kA/m and a frequency of 100 ​kHz was safely applied to the brain in multiple stages [235]. The advantage of MHT for glioma treatment is that it can guide treatment more precisely due to the tumor tissue specificity of INPs. It can also enhance treatment safety due to the lower destruction of intracranial glioma at lower temperatures [236,237]. More importantly, it has the non-invasive feature with unrestricted tissue penetration. For instance, virtually, all of the AMF energy at 400 ​kHz can be targeted to sites of MNPs accumulation, even penetrating up to 15 ​cm [238].

Sawant and colleagues [239] synthesized pH-sensitive alloy-drug nanoconjugates (SPANs) based on a composite of Fe and Pt that have excellent heating ability and can be excited by both AMF and NIR lasers (Fig. 12C). The pH-responsive drug release can help overcome drug and metal damage to normal cells. The glioma cell temperature was increased by 8–11 ​°C under the dual heating mode at 335.2 ​Oe AMF and 1064 ​nm laser irradiation. On account of the higher absorbance in the NIR-II window deriving from the Fe^2+^–Fe^3+^ transition in SPANs, the heating under 1064 ​nm irradiation was higher than under 808 ​nm irradiation. Furthermore, Fe-based alloy nanoparticles catalyzed by the Fenton and Haber-Weiss reactions may generate ROS to induce tumor death. Summarily, the work demonstrate that SPANs excited by both AMF and NIR lasers enhance the suppression of U87MG cell viability.

A magnetosome chain is an iron oxide nanoparticle (IONP) synthesized by magnetotactic bacteria, which can replace chemically synthesized IONP and further improve the therapeutic effect. Alphandéry and colleagues [240] fabricated magnetosomes-poly-l-lysine (M-PLL) by removing potentially toxic organic bacterial residues and coating magnetosome mineral core surfaces with poly-l-lysine to achieve complete biocompatibility. The anti-glioma efficacy of M-PLL was compared with that of chemically synthesized IONPs currently used in magnetic hyperthermia (Fig. 12D). When GL-261 (murine glioma cell line) subcutaneous glioma tumors were exposed to AMF at a frequency of 198 ​kHz and 11–31 ​mT, their temperature could reach 43–46 ​°C within 30 ​min. This shows that M-PLL can achieve better therapeutic effects at a very low magnetic field and energy consumption. More importantly, MHT induced by M-PLL ​+ ​AMF can completely ablate tumors or inhibit tumor growth. In conclusion, MHT for GBM has been improved by the use of biocompatible magnetosomes.

MNPs for glioma MHT have tumor specificity and long-lasting therapeutic effects. Compared with PTT, the penetration depth of MHT is deeper. Moreover, due to the optical catalytic activity of some magnetic metal NPs, the combination of MHT with phototherapy can increase the therapeutic outcome of glioma. Although MHT has been shown to be effective *in vitro*, in animal models, and in human experiments, there are still some challenges in becoming a standard of treatment for gliomas, such as accurate thermometry within the tumor mass and precise tumor heating.

### 6.3 Multifunctional inorganic nanoplatforms for glioma diagnosis or treatment

With the development of nanotechnology, increasing numbers of multifunctional INPs have arisen for tumor diagnosis and therapy. This presents a new perspective for establishing efficiency and precision-guided multifunctional glioma treatment platforms. Representative examples of INPs applied to the integrated diagnosis and treatment of glioma in the past decade are summarized in Table 4, which can provide optimal preparation strategies for multifunctional nanoplatforms as required. In the applications described above, many INPs have been modified or doped according to the functions of different dominant elements. This enables nanocomposites to obtain three or more kinds of multi-functional diagnosis or treatment characteristics, thereby improving the efficiency of integrated glioma treatment.

**Table 4:** Summary of glioma diagnosis and treatment based on inorganic nanoplatforms.

Inorganic nanoplatform		Size (nm)	Cell type	Cell safety concentration (Incubation time)	Animal tumor model	Application(s)	Ref.
Au	TAT-Au NP	5.9 ​± ​2.1	U87	None	Intracranial U87 mice model	MRI	[58]
	DOX@CMXG @AuNPs	8–10	LN-299	10 ​μg/mL (24 ​h)	None	FLI	[247]
	AuNPs	20	C6	None	None	FLI	[248]
	PEI-based Au NPs	151.0 ​± ​25.4	C6	None	Subcutaneous C6 rats model	CT, RT	[72]
	RGD-[64Cu] Au NR	L: 25.1 ​± ​2 W:8.0 ​± ​0.5	U87	None	Subcutaneous U87 mice model	PET, PTT (808 ​nm)	[249]
	AuNDIPs	282 ​± ​15	C6	500 ​μg/mL (24 ​h)	None	PTT/PDT (808 ​nm) /CHEMO	[211]
	GC-pep@SiNC -AuNC	160	U87	5 ​μg/mL (6 ​h)	Intracranial U87 mice model	PDT (785 ​nm)	[250]
	SNAs	13	U87	5 ​nM (24 ​h)	Intracranial U87 mice model	GT	[251]
	SNAs	13 ​± ​1	U87 and GIC-20	None	Intracranial U87/GIC-20 mice model	GT	[252]
	RDGNR/shRNA	L: 50 W: 10	U87	25 ​μg/mL (48 ​h)	Subcutaneous U87 mice model	GT	[253]
	Au PENPs	24.2	U87	3000 ​nM (24 ​h)	Subcutaneous U87 mice model	GT	[254]
	D&H-A-A&C	38.1 ​± ​1.6	C6	None	Intracranial C6 mice model	IMT/CHEMO	[255]
	Au–OMV	42	G261, bEND.3 and C8D1A	2 ​μg/mL (24 ​h)	Subcutaneous/Intracranial G261 mice model	IMT/RT	[101]
	cRGD–HN–DOX	90	U87	10 ​μg/mL (4 ​h)	Subcutaneous U87 mice model	CHEMO	[256]
	An-PEG-DOX-AuNPs	39.9	C6	None	Intracranial C6 mice model	CHEMO	[64]
	AuNPs-DC-RRGD	55.9	C6	None	Intracranial C6 mice model	CHEMO	[171]
	GNP-UP-Cis	7	U87, U251, T98G and U138	None	Intracranial U251 mice model	CHEMO/RT	[124]
	Au PENPs-CTX	4.4 ​± ​1.3	C6	200 ​μM (24 ​h)	Subcutaneous/Intracranial C6 mice/rats model	CT, RT	[72]
	BSA-AuNPs	18	U87	0.3 ​mg/mL (24 ​h)	Subcutaneous U87 mice model	RT	[106]
	FA-AuNCs	5.5 ​± ​0.4	C6 and L929	160 ​μg/mL (24 ​h)	Intracranial C6 rats model	RT	[257]
	DNA-AuNPs	4.5	U251	None	None	RT	[258]
Ag	Ag_2_S QDs	5.4 ​± ​0.3	MDA-MB-468 and U87	None	None	FLI	[259]
	AgNPs	21.2 ​± ​0.31	C6	None	Intracranial C6 rats model	RT	[260]
	Ag-PNP-CTX	114 ​± ​2	U87 and T98	None	Intracranial U87 mice model	RT	[261]
Cu	Cu_2-x_Se NPs	3 ​± ​0.3	None	None	None	PAI/PET	[79]
Cd	CdTe/CdS QDs	4.05 ​± ​0.3	U251	None	None	FLI	[262]
	QD-MPA	None	293 ​T, HUVEC, MCF7, U87 and SW620	None	Subcutaneous HT1080/MCF-7 mice model	FLI	[73]
	CdSe QDs/CdSeNPls	22 ​± ​2/33 ​± ​8	C6	None	None	FLI	[263]
	NGR-PEG-QDs	10	BCECs, astrocytes and C6	20 ​nM (96 ​h)	Intracranial C6 rats model	FLI	[74]
	QD-Apt	20	U87 and HUVEC	None	Intracranial U87 mice model	FLI	[75]
Zn	ZnS-QDs	3.6	U-87 MG, HEK293T	None	None	FLI, CHEMO	[162]
	CdSSe/ZnS QDs	12.97 ​± ​4.62	U87 and U251	20 ​μg/mL (48 ​h)	None	GT	[194]
	ZnS@CMC-DOX	3.6	HEK 293 ​T and U87	None	None	FLI, CHEMO	[162]
Ti	Au NR@aTiO_2_ @UCNPs	∼200	U87	120 ​μg/mL (24 ​h)	Subcutaneous U87 mice model	PTT/PDT (808 ​nm)	[213]
Mo	MoS2 Nanosheets	181 ​± ​3	U87	1 ​mg/mL (1.5 ​h)	Subcutaneous U87 mice model	PAI	[149]
La (Er)	NaErF4:Ce@NaAF4@NaLuF4	17.9 ​± ​0.9	U87	800 ​μg/mL (24 ​h)	Subcutaneous/Intracranial U87 mice model	FLI	[76]
	La2O3 NPs	<100	RN1, GBML1, WK1, PDCL G53 and NHA	None	None	RT	[190]
Gd	PFG-Ce6-Gd NPs	130	C6	1.25 ​μg/mL (12 ​h)	Subcutaneous C6 rats model	MRI	[96]
	ANG/PEG-UCNPs	17.2 ​± ​0.5	U87	1000 ​μg/mL (24 ​h)	Intracranial U87/BCECs mice model	MRI/FLI	[264]
Fe	Pac-MNPs	202 ​± ​3.7	U87	None	Intracranial U87 rats model	MRI	[265]
	IONPs	33.24	U87	2 ​mg/mL (2 ​h)	Subcutaneous U87 mice model	MRI	[125]
	PDNCs	100–120	RG2	1000 ​μg/mL (24 ​h)	Intracranial RG2 rats model	MRI/FLI	[66]
	GPEI-Fe NPs	110	9 ​L	45 ​μg/mL (3 ​h)	Intracranial 9 ​L rats model	MRI	[266]
	CS-DX-SPIONs	55	U87 and C6	10 ​μg/mL (24 ​h)	Intracranial C6 rats model	MRI	[267]
	SPIO@AuNP	82	U87	4 ​μg/mL (48 ​h)	Intracranial U87 mice model	MRI/PAI	[268]
	Fe@Au NPs	164 ​± ​44	HFF-1 and C6	100 ​μg/mL (24 ​h)	Subcutaneous C6 mice model	MRI/CT, PTT (1064 ​nm)	[215]
	SPIO@DSPE-PEG/DOX/ICG NPs	22.9	U251, C6 and HUVECs	100 ​μg/mL (72 ​h)	Intracranial C6 rats model	MRI/FLI, CHEMO	[246]
	IONPs	L: 20 ​± ​5 W: 17 ​± ​4	GL-261	None	Subcutaneous GL-261 mice model	MHT	[240]
	Fe_3_O_4_ @Au–C225 MNPs	46	U251	None	Subcutaneous U251 mice model	MHT	[94]
	M-PLL and IONP	17–20	3T3 and U87	None	Intracranial U87 mice model	MHT	[269]
	MCNP-ATAP	46.8 ​± ​2.3	U87 and MDA-MB-231	50 ​μg/mL (48 ​h)	None	MHT	[270]
Si	MSNs	2.9	U87	400 ​μg/mL (48 ​h)	None	CT	[271]
	SiNPs	45	None	3 ​mg/mL (24 ​h)	None	FLI	[272]
	MSNs	88.5 ​± ​2.2	U87MG	None	Subcutaneous U87 mice model	PET	[139]
	pSiNPs	L: 7.5 W: 2.5	U87 and 4T1	None	Subcutaneous U87 mice model	GT	[196]
	DOX@MSN–SS–iRGD&1 ​MT	50	GL261	None	Intracranial GL261 mice model	IMT/CHEMO	[70]
	DOX@MSN	20/40/80	U87, U251, C6 and CHEM-5	None	None	CHEMO	[48]
	CPT@MSN-hyd-DOX	2.9	U87	400 ​μg/mL (48 ​h)	None	CHEMO	[271]
	MNP-MSN-PLGA-Tf NPs	∼150	bEND.3 and U87	100 ​μg/mL (96 ​h)	Intracranial U87 mice model	CHEMO	[67]
	DOX-MNP-MSN-PF-127-Tf	110	U87	1 ​μg/mL (96 ​h)	None	CHEMO	[68]
	VPA-MSNs	L: 180 W: 120	C6 and U87	100 ​mg/mL (24 ​h)	None	RT	[273]
GPE	GSPID	50–250	U251	50 ​μg/mL (2 ​h)	None	PTT (808 ​nm) /CHEMO	[274]
	GQDs/DOX @CCM	130 ​± ​10	BV2, GMI-R1 and rat astrocyte	200 ​μg/mL (24 ​h)	None	PTT (808 ​nm) /CHEMO	[100]
C	NCDDG	30	U87	256 ​μg/mL (24 ​h)	Intravenous U87 mice model	MRI/FLI	[275]
	O-MWNTs-PEG-ANG	10	C6	100 ​μg/mL (24 ​h)	Intracranial C6 mice model	FLI	[71]
	CD-Asp	2.28 ​± ​0.42	C6	None	Intracranial C6 mice model	FLI	[47]
	HCCDs	6–8	U87	800 ​μg/mL (24 ​h)	Intracranial U87 mice model	FLI/PAI, PTT (808 ​nm)	[242]
	CNT	None	U251, U87, LN229 and T98G	3 ​μg/mL (72 ​h)	Subcutaneous U251 mice model	PTT (NIR)	[276]
	CNT	8–15	U87, U373, NHA and D54	100 ​μg/mL (24 ​h)	None	PTT (970 ​nm)	[277]

∗L represents for Length; W represents for Width; CHEMO represents for chemotherapy; RT represents for radiotherapy; GT represents for gene therapy; IMT represents for immunotherapy; FLI represents for fluorescence imaging.

#### 6.3.1 The glioma multimodality molecular imaging based on INPs

Multimodality molecular imaging combines two or more imaging techniques, integrating their benefits while overcoming the limitations of a single imaging modality, so as to offer sufficient information for accurate diagnosis. Advances in the design and synthesis of inorganic nanoplatforms will offer the ability to produce novel multimodality imaging probes, which can target glioma and reflect more pathophysiological details. Multimodality molecular imaging based on INPs can be tailored as necessary, such as through the assembly of inorganic elements with diverse properties and the encapsulation of molecules with varying functions.

**Fig. 13:**
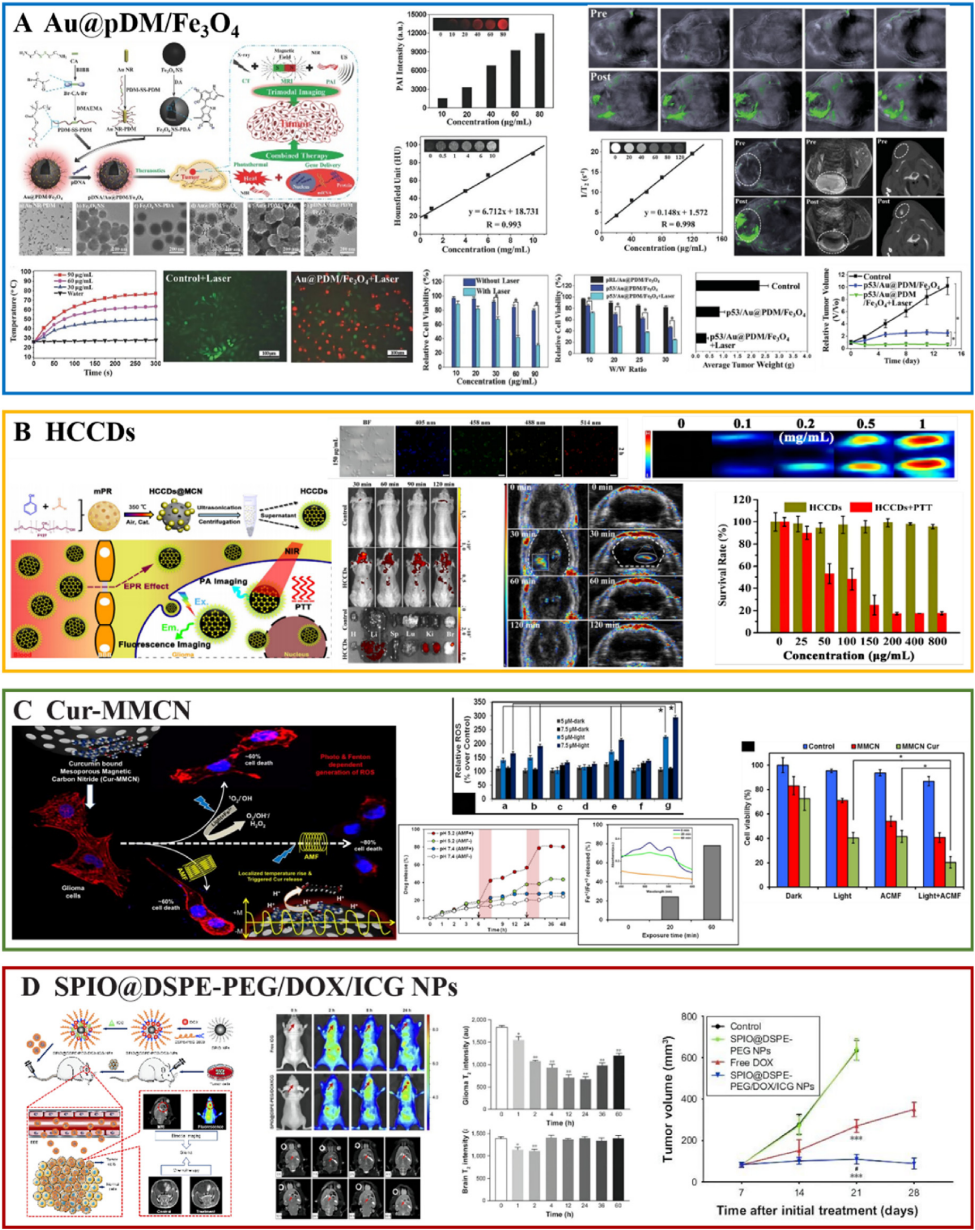
**Multifunctional inorganic nanoplatforms for combined glioma diagnosis or treatment.** A. Au@PDM/Fe_3_O_4_; B. HCCDs; C. Cur-MMCN; D. SPIO@DSPE-PEG/DOX/ICG NPs. Panel A is adapted with permission from Ref. [241], copyright 2016 Materials Views. Panel B is adapted with permission from Ref. [242], copyright 2018 ACS Applied Materials & Interfaces. Panel C is adapted with permission from Ref. [245], copyright 2019 ACS biomaterials science & engineering. Panel D is adapted with permission from Ref. [246], copyright 2019 International Journal of Nanomedicine.

Xu and colleagues [241] constructed multifunctional hierarchical nanocomposites (pDNA/Au@PDM/Fe_3_O_4_) for trimodal PAI/MRI/CT diagnostic methods through a consecutive electrostatic assembly technique (Fig. 13A). Au@pDM/Fe_3_O_4_ is based on ternary assemblies of negatively charged Fe_3_O_4_ cores (Fe_3_O_4_-PDA), polycation-modified Au nanorods (Au NR-pDM) and polycations. In terms of optical imaging, this nanocomposite can be used for PAI because of the combined NIR absorbance properties of Fe_3_O_4_-PDA and Au NR-pDM, which can greatly enhance optical absorption by tissue and reflect the characteristics of tumor tissue. Besides, Fe_3_O_4_ NPs and Au NPs have been demonstrated to be promising T2-weighted MRI and CT contrast agents. *In vitro* and *in vivo* assay results suggest that the pDNA/Au@PDM/Fe_3_O_4_ should be one promising trimodal contrast agent for PAI/MRI/CT of tumors.

In addition to the hierarchical nanocomposites mentioned above, Huang et al. [242] developed multicolour highly crystalline carbon nanodots (HCCDs) for glioma dual-modality molecular imaging (Fig. 13B). HCCDs were synthesized using F127 and phenol resin as a carbon source to form a mesoporous carbon template via an in-situ solid-state transformation technique. The HCCDs contain a highly crystalline carbon nanocore and a hydrophilic surface, thereby providing both intense photoacoustic as well as adjustable fluorescence emissions. The potential to use HCCDs for dual-modal-imaging-guided PTT of gliomas *in vitro* and *in vivo* was verified. According to these observations, a visualized PTT for gliomas can be guided by deep space-resolved PAI and sensitive fluorescence imaging, where the timing and location of NIR irradiation can be adjusted to minimize side effects to normal tissue following the injection of HCCDs.

In brief, the combination of multiple diagnostic approaches can be achieved as needed by using appropriate inorganic components and construction strategies. Although this field is still in its infancy, multimodality molecular imaging based on INPs has exciting features in glioma diagnosis.

#### 6.3.2 The glioma synergistic therapy based on INPs

As described above, Xu [241] constructed pDNA/Au@PDM/Fe_3_O_4_ for synergistic gene therapy and PTT (Fig. 13A). Positively charged Au@pDM/Fe_3_O_4_ is also able to compound plasmid DNA into pDNA/Au@pDM/Fe_3_O_4_ and efficiently mediate gene therapy. It is well known that the p53 gene is a tumor-suppressor and could be applied for clinical gene therapy [243,244]. Au@PDM/Fe_3_O_4_ can be employed to deliver plasmid p53 (cloned with p53 genes) for gene therapy. Furthermore, Au@PDM/Fe_3_O_4_ produced a great temperature change of 50.1 ​°C after laser irradiation for 5 ​min, indicative of excellent photothermal conversion performance. Glioma C6 cells incubated with Au@PDM/Fe_3_O_4_ were irradiated with an 808 ​nm laser, displaying substantially lower cell viability. *In vitro* molecular mechanism experiments demonstrated that cells incubated with p53/Au@PDM/Fe_3_O_4_ exhibited distinctly lower viability than pRL/Au@PDM/Fe_3_O_4_ (control), and can be further eliminated by laser irradiation. Likewise, a p53/Au@PDM/Fe_3_O_4_ nanoplatform exhibited strong tumor growth inhibition ability *in vivo* antitumor therapy combining PTT and p53 gene therapy. These results demonstrate that the combination of gene therapy and PTT based on p53/Au@PDM/Fe_3_O_4_ nanoplatform can obviously increase glioma therapeutic effects.

Another example of glioma synergistic therapy based on INPs is a mesoporous magnetic nanohybrid as a combination platform for chemotherapy, PDT and MHT (Fig. 13C) [245]. The mesoporous magnetic nanohybrid was functionalized with 14 ​wt% carbon nitride (CN) and loaded with Cur (Cur-MMCN). Cur-MMCN is formed by means of physical adsorption between the extended planar structure of Cur and the 2D architecture of CN. The combination of acidic pH and AMF could trigger the release of Cur from MMCN. CN based nanomaterials have emerged as a new class of photocatalysts for PDT. Cur-MMCN increased ROS by 350% at a 7.5 ​μM concentration under blue LED light irradiation compared to control groups. In addition to the photocatalytic anti-glioma effect of Cur-MMCN, the intrinsic presence of H_2_O_2_ in glioma cells could also assist in the generation of high reactive hydroxyl and superoxide radicals catalyzed by Fe^2+^ ions through the Fenton reaction. Cur-MMCN activated with blue light effectively killed ∼60% of cells, while cell viability was further reduced to ∼15% when co-exposed to AMF. The caspase 3/7 apoptosis assay showed that a combination of mild MHT and high oxidative stress is capable of triggering the apoptotic pathway leading to the highest level of glioma cell death. Summarily, the combination of Cur-MMCN, blue light and AMF achieves better glioma therapeutic outcomes.

#### 6.3.3 Combined glioma diagnosis and treatment based on INPs

One of the classic works on a multifunctional inorganic nanoplatform for synergistic glioma diagnosis and treatment is imaging-mediated chemotherapy. Shen et al. [246] constructed a novel multifunctional drug delivery system (SPIO@DSPE-PEG/DOX/ICG NPs), which combines real-time MR/fluorescence imaging and chemotherapy (Fig. 13D). Hydrophobic superparamagnetic iron oxide nanoparticles (SPIONPs) stabilized with oleylamine and oleic acid were prepared by a thermal decomposition method. DOX-loaded NPs were coated with DSPE-PEG 2000 using a classic thin-film hydration method. The amphiphilic structure of ICG enabled it to be readily loaded onto the phospholipid layer of the NPs. SPIO can be used as an MRI contrast agent that has excellent spatial resolution but poor sensitivity. Therefore, the combination of SPIO with the highly sensitive fluorescent imaging agent ICG could achieve great dual-modality imaging results. The fluorescence signal of free ICG was detectable in mice only 2 ​h after SPIO@DSPE-PEG/DOX/ICG NPs injection, after which the signal gradually faded. After 24 ​h, the majority of the signals was concentrated in the tumor tissue, which indicated that the NPs could be blocked mainly in tumor tissues due to strong EPR effects. Furthermore, the MR imaging data showed that the multifunctional NPs remained in the tumor for at least 60 ​h, while the non-tumor brain areas returned to the pre-injection level after 4 ​h. Subsequently, they investigated the effect of chemotherapy using SPIO@DSPE-PEG/DOX/ICG NPs in orthotopic glioma-bearing rats, with tumor sizes monitored in real-time using MRI. A rapid rate of tumor growth was observed in brain MR images of control rats, whereas rats treated with SPIO@DSPE-PEG/DOX/ICG NPs showed inhibited tumor growth. The current results provide useful information for the design and development of multifunctional nanocomposites for the diagnosis and treatment of glioma based on appropriate assemblies of various INPs and polycations.

## 7 Conclusion and future perspectives

In this review, we extensively discussed the last decade's advances in developing inorganic nanomaterials for the treatment and diagnosis of glioma. As the treatment of intracranial glioma is more difficult than treatment in other organs, there are also few suitable nanomaterials. The design and preparation of theranostic nanomaterials are based on the disease characteristics of glioma. It is also necessary to consider the characteristics of the BBB in the different stages of glioma and to enhance “sequential targeting” across the BBB and glioma tissues by connecting targeting groups. The choice of targeting groups is also important. As damage to the BBB occurs in different stages of glioma, different targeting groups could be selected. In the early stage when the BBB is relatively complete, bipolar targeting could be considered, while in the late stage when the BBB is completely destroyed, suitable ligand molecules for targeting gliomas should be considered (specific molecules are summarized in Table 2).

As proliferating gliomas have unclear boundaries, clear imaging techniques are extremely important. Many inorganic nanomaterials can be constructed by doping and other methods to achieve multimodal imaging ability, which can improve diagnostic accuracy, identification of glioma margins and surgical resection [278]. Also, targeting efficiency can be improved by connecting different active targeting groups. The choice of imaging tool depends on how different materials respond to excitation conditions. In terms of treatment, the main functions of INPs are as carriers to deliver chemotherapy drug, immune adjuvants, and siRNAs that go into the brain. The intrinsic physical properties of inorganic nanomaterials, such as their laser and nuclear magnetic response characteristics, can also be used to improve imaging sensitization and chemotherapy efficacy, or to regulate drug duration and release efficiency. At the same time, because of the increasing number of active targeting groups, much work has been repeatedly verified to obtain some efficient targeting groups, such as low-density lipoprotein receptor-associated protein-1,^64^ hyaluronic acid [279], Tf [280] and rituximab [281].

Gd-based contrast agents have been used in the clinical diagnosis of glioma. Gd-based AGuIX nanoparticles have also been reported to have achieved promising results in Phase I clinical trials. In one trial, 13 out of 14 patients with evaluable brain metastases showed clinical benefit [282]. Moreover, a non-randomized clinical trial has confirmed that fluorescence-guided sentinel lymph node (SLN) biopsy based on ultrasmall, molecularly targeted core-shell silica nanoparticles is feasible and safe for the treatment of head and neck melanoma [283]. Research into nanomaterials is also accelerating the pace of clinical applications; however, there are still several important issues that need to be addressed. For instance, the type, size, shape, surface charge, modification and other characteristics of nanoparticles may affect their efficacy *in vivo* and influence the structure of the BBB. These factors may not only affect cytotoxicity but also the distribution and metabolism of nanoparticles, which are associated with long-term toxicity to the brain and other parts of the organism. Furthermore, systematic evaluation of the effects on brain function, such as changes in learning, cognitive ability, BBB integrity and permeability, is needed.

In addition to biocompatibility, the dosage of medications required to achieve the desired effects must be considered due to the high permeability of nanoparticles. Surface functional modifications can reduce cytotoxicity and improve biocompatibility. Different surface charges and surface modifications can generate different consequences. In general, depending on electrostatic interactions, positively charged INPs are more easily absorbed by cell membranes than negatively or neutrally charged INPs, which have longer blood circulation times because they have difficulty binding to proteins or cells in the blood [[284], [285], [286], [287]]. For gold nanomaterials, chemical modification of the surface may produce different degrees of immune stimulation; this property can be applied in immunotherapy [288]. Thus, we should consider their intrinsic properties to achieve the desired effect when designing nanomaterials. Additionally, the mechanism of cell death induced by some INPs is increased intracellular ROS levels, as well as involving apoptosis and autophagy pathways. The other potential mechanism of treating glioma based on INPs is still unclear and needs further exploration.

Moreover, extrinsic physical techniques, especially lasers, can be used to enhance chemotherapy and phototherapy and improve the quality and sensitivity of *in vivo* PAI and fluorescence imaging. In addition, the excellent photostability and long half-life of INPs make them ideal for phototherapy. To improve skull penetration efficiency, research on how inorganic nanocomplexes respond to NIR-II lasers is providing new strategies for the optical diagnosis and treatment of glioma. Considering intracranial safety, the commonly used methods are low-injury hypothermia PTT and PDT. However, whether the ROS over-produced by PDT has side effects on normal tissues still needs to be studied, so as to determine the optimal ROS production rate and achieve the best therapeutic effect. Besides, evaluation of the long-term toxicity of degraded nanocomposites *in vivo* should not be ignored.

In conclusion, the current progress and issues in glioma theranostics have created demands for a new generation of nanomaterials. The neurotoxicity of multifunctional nanomaterials still warrants further exploration. INPs based new generation nanoplatform is also expected to provide more effective diagnosis and treatment of glioma and extend human life.

## Ethics approval

This study does not contain any studies with human or animal subjects performed by any of the authors.

## Declaration of competing interest

The authors declare that they have no known competing financial interests or personal relationships that could have appeared to influence the work reported in this paper.

## Data Availability

Data will be made available on request.
